# Herbal terpenoids activate autophagy and mitophagy through modulation of bioenergetics and protect from metabolic stress, sarcopenia and epigenetic aging

**DOI:** 10.1038/s43587-025-00957-4

**Published:** 2025-09-24

**Authors:** Gabriele Civiletto, Dario Brunetti, Giulia Lizzo, Kamila Muller, Guillaume E. Jacot, Ioanna Daskalaki, Federico Sizzano, Minji Huh, Ivano Di Meo, Maria Nicol Colombo, José L. Sanchez-Garcia, Bertrand J. Bétrisey, Alix Zollinger, Patricia Lino, Christopher Neal, Anne-Laure Egesipe, Joy Richard, Myriam Chimen, Aurélie Hermant, Benjamin Brinon, Lorane Texari, Sylviane Metairon, Mohammed Adnan Qureshi, Dhaval S. Patel, Siva A. Vanapalli, Marco Malavolta, Arwen W. Gao, Amelia Lalou, Mauro Provinciali, Fiorenza Orlando, Valeria Tiranti, Robert T. Brooke, Steve Horvath, Johan Auwerx, Jerome N. Feige, Philipp Gut

**Affiliations:** 1 Nestlé Institute of Health Sciences, Nestlé Research, Lausanne, Switzerland; 2 Health, Nutrition and Care, DSM-Firmenich, Kaiseraugst, Switzerland; 3 Unit of Medical Genetics and Neurogenetics, Fondazione IRCCS Istituto Neurologico Carlo Besta, Milan, Italy; 4 Department of Clinical Sciences and Community Health, Dipartimento di Eccellenza 2023–2027, University of Milan, Milan, Italy; 5 Laboratory of Integrative Systems Physiology, Interfaculty Institute of Bioengineering, École Polytechnique Fédérale de Lausanne, Lausanne, Switzerland; 6 Nestlé Institute of Food Safety and Analytical Sciences, Nestlé Research, Lausanne, Switzerland; 7 NemaLife Inc., Lubbock, TX USA; 8 Texas Tech University, Lubbock, TX USA; 9 Advanced Technology Center for Aging Research and Geriatric Mouse Clinic, IRCCS INRCA, Ancona, Italy; 10 Department of Clinical and Molecular Sciences (DISCLIMO), Università Politecnica delle Marche, Ancona, Italy; 11 Experimental Animal Models for Aging Unit, Scientific Technological Area, IRCCS INRCA, Falconara Marittima, Italy; 12 Epigenetic Clock Development Foundation, Los Angeles, CA USA; 13 Department of Human Genetics, David Geffen School of Medicine, University of California, Los Angeles, Los Angeles, CA USA; 14 Altos Labs, San Diego, CA USA; 15 EPFL School of Life Sciences, Ecole Polytechnique Fédérale de Lausanne, Lausanne, Switzerland; 16 Present Address: Amsterdam Gastroenterology, Endocrinology, and Metabolism, Amsterdam UMC, University of Amsterdam, Amsterdam, the Netherlands

**Keywords:** Mitophagy, High-throughput screening, Metabolic disorders, Experimental models of disease, Ageing

## Abstract

Small molecular food components contribute to the health benefits of diets rich in fruits, vegetables, herbs and spices. The cellular mechanisms by which noncaloric bioactives promote healthspan are not well understood, limiting their use in disease prevention. Here, we deploy a whole-organism, high-content screen in zebrafish to profile food-derived compounds for activation of autophagy, a cellular quality control mechanism that promotes healthy aging. We identify thymol and carvacrol as activators of autophagy and mitophagy through a transient dampening of the mitochondrial membrane potential. Chemical stabilization of thymol-induced mitochondrial depolarization blocks mitophagy activation, suggesting a mechanism originating from the mitochondrial membrane. Supplementation with thymol prevents excess liver fat accumulation in a mouse model of diet-induced obesity, improves *pink-1*-dependent heat stress resilience in *Caenorhabditis elegans*, and slows the decline of skeletal muscle performance while delaying epigenetic aging in SAMP8 mice. Thus, terpenoids from common herbs promote autophagy during aging and metabolic overload, making them attractive molecules for nutrition-based healthspan promotion.

## Abstract (web summary)

Using a zebrafish-based screen, Civiletto and colleagues identify the herb-derived metabolites thymol and carvacrol as activators of autophagy and mitophagy, demonstrating their therapeutic potential in *C. elegans* and mouse models.

## Main

Despite an extraordinary increase in lifespan over the past 170 years^1^, the prevalence of chronic diseases among the rapidly growing population of older adults remains high due to environmental, lifestyle and nutritional factors^2^. Among these, inadequate nutrition makes a major contribution to the onset and progression of noncommunicable diseases and mortality. The intake of excess calories, as well as the overconsumption or underconsumption of specific food and nutrient groups, has been associated with a wide range of negative health outcomes^3^. Prevention through nutritional approaches, sometimes referred to as ‘food as first medicine’, is therefore gaining importance in lowering the burden of noncommunicable diseases^4^. Beyond recommendations for adequate portion sizes and balanced consumption of nutrient-dense foods, specific dietary patterns are effective tools for disease prevention. For instance, caloric restriction and regimens under the umbrella term of intermittent fasting, such as alternate-day fasting, 5:2 fasting and time-restricted eating, show promise in promoting healthy aging not only through weight loss but also through other mechanisms independent of changes in weight^5–8^. At the cellular level, various molecular adaptations promote the health benefits of caloric restriction and intermittent fasting, among which the activation of autophagy is believed to be a principal mechanism explaining their geroprotective effects^9^. Autophagy prevents proteotoxicity and cellular damage by recycling protein aggregates and dysfunctional organelles, leading to improved organism resilience against metabolic challenges and other age-related cellular stressors^10^. The requirement for intact autophagy to mediate the benefits of caloric restriction in extending healthspan and lifespan has been demonstrated by genetic loss-of-function experiments in a wide range of experimental models (including yeast, worms and flies) carrying deletions in genes related to autophagy^11^. Similar findings have been confirmed in mammals. For example, genetic ablation of autophagy in the liver, adipose tissue, skeletal muscle or hypothalamic proopiomelanocortin neurons of mice abolishes the system-wide beneficial effects of intermittent fasting, suggesting a conserved role of autophagy for healthy aging in mammals^12^.

The uptake of dietary patterns that activate autophagy is complicated by the need for low-calorie intake over an extended period before autophagy is efficiently induced. In addition, the efficacy with which fasting activates autophagy shows a large variability across individuals^13^. To circumvent these challenges, small molecules could be used to mimic the benefits of fasting and caloric restriction while following a diet with normal or only slightly reduced caloric intake^14^. Such a strategy should ideally be based on food-grade ingredients that activate autophagy and can be used in a dietary context for preventive health before drugs are medically indicated. For example, spermidine, urolithin A and resveratrol have been shown to provide broad health benefits across organ systems, with a remarkable level of conservation across species^15–19^. However, the number of natural bioactive compounds that can be safely used at efficacious levels in food and beverage formats remains small, limiting the potential to promote autophagy as part of diets that can be widely adopted for disease prevention.

In this study, we developed and deployed a rapid testing approach in zebrafish as a suitable whole-organism model for small-molecule discovery, with the aim of finding food-grade autophagy activators^20^. Zebrafish have been used to measure autophagy in vivo, providing a vertebrate model that recapitulates organismal complexity^21,22^. In practice, the use of zebrafish for the discovery of autophagy modulators has been limited by the need for microscopy and the quantification of autophagy in individual animals^23,24^. In this study, we established an in vivo workflow for the discovery of autophagy modulators using a transgenic construct in which ZsGreen is tagged to the autophagosome-localized protein LC3 (encoded by *map1lc3*), combined with high-content image acquisition and analysis in live zebrafish. We tested a library of natural bioactive molecules and identified thymol and carvacrol, major components of thyme and oregano, as autophagy inducers in zebrafish, mice and human cells. Mechanistically, thymol transiently lowers the mitochondrial membrane potential (MMP), triggering a cellular stress response that promotes the recycling of damaged mitochondria. Thymol supplementation reduces hepatic fat content in a mouse model of diet-induced obesity, delays age-related phenotypes in worms, and lowers epigenetic age in conjunction with improved muscle performance in a naturally occurring mouse strain affected by premature mobility decline.

## Results

### High-content workflow to monitor autophagic flux in vivo

**Fig. 1:**
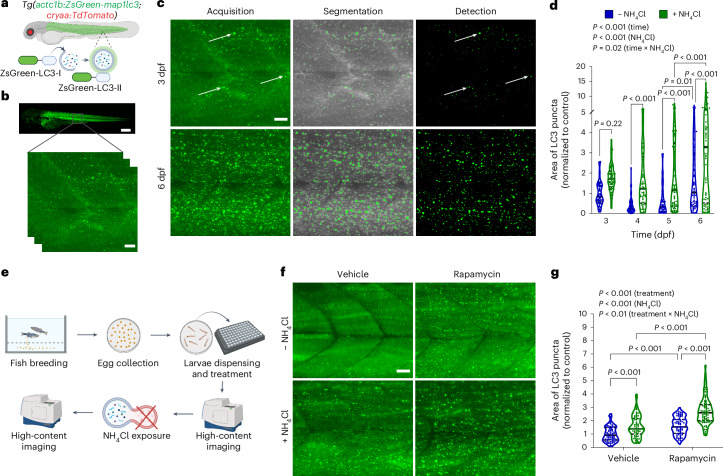
Development of high-content imaging-based assay to quantify autophagic flux in zebrafish larvae. **a**, Schematic of the transgenic *Tg(actc1b:ZsGreen-map1lc3;cryaa:TdTomato)* zebrafish line for live monitoring of autophagy. **b**, Representative fluorescence image of transgenic ZsGreen-LC3 zebrafish larvae at 3 dpf, acquired at 4× (top) and 20× (bottom) magnifications. **c**, Detection of LC3-positive vesicles through semiautomated high-content imaging in transgenic larvae at 3 dpf (top) and 6 dpf (bottom). After acquisition, images were processed to locate objects corresponding to LC3 puncta (segmentation). Arrowheads indicate ZsGreen accumulation in autophagosomes in response to autophagy activation. The resulting objects were identified and counted as described in Extended Data Fig. 1b. **d**, Measurement of autophagic flux in zebrafish larvae by relative quantification of the total area of LC3 puncta normalized to control before and after 4 h of exposure to 100 mM NH_4_Cl (− NH_4_Cl: 3 dpf, *n* = 72; 4 dpf, *n* = 59; 5 dpf, *n* = 60; 6 dpf, *n* = 69; + NH_4_Cl: 3 dpf, *n* = 75; 4 dpf, *n* = 61; 5 dpf, *n* = 68; 6 dpf, *n* = 72). **e**, Workflow for rapid testing of small molecules in 96-well microplates to identify modulators of autophagy. **f**, Representative images of transgenic zebrafish larvae at 3 dpf treated for 16 h with 1 µM rapamycin (top), followed by treatment with NH_4_Cl (bottom). **g**, Quantification of autophagic flux in zebrafish larvae treated with rapamycin (3 dpf; vehicle −/+ NH_4_Cl, *n* = 68; rapamycin − NH_4_Cl, *n* = 67; rapamycin + NH_4_Cl, *n* = 71) and normalized to control (vehicle, 1% DMSO). Violin plots in **d** and **g** report the median (solid lines) and quartiles (dotted lines). Data were pooled from three independent experiments and analyzed by two-way ANOVA followed by Sidak multiple comparisons. Scale bars, 100 µm (**b** top) and 20 µm (**b** bottom, **c** and **f**). Panels **a** and **e** created with BioRender.com. Source data

**Extended Data Fig. 1:**
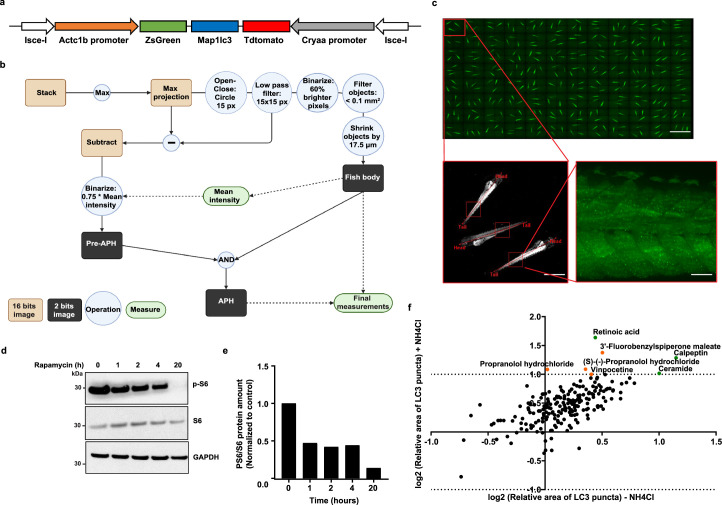
Zebrafish autophagy reporter validation and high content screening. **a** Graphical representation of the construct used to generate transgenic *Tg(actc1b:ZsGreen-map1lc3;cryaa:TdTomato)* zebrafish to monitor autophagy. **b** Schematic workflow for image analysis of transgenic zebrafish larvae. 20X (0.75 NA) objective Z stacks (26 images, 2 µm apart) of the bodies were acquired in FITC (536 nm) channel using automated microscope (ImageXpress, Molecular Devices) and a 60 µm pinhole spinning disk. **c** Image acquisition of transgenic zebrafish larvae in 96 microwell plate. Scale bars, 10 mm (top); 1 mm (bottom-left); 100 µm (bottom-right) **d** Western blot images showing time-course experiment of inhibition of mTORC1 activity in 3 dpf zebrafish larvae treated with 1 µM rapamycin. **e** Densitometric quantification of western blot. **f** Scatterplot showing autophagy flux in zebrafish larvae treated with 240 selected chemicals from TOCRIS library at 10 µM (3 dpf, n = 12 per compound). Dashed lines indicate 1-fold log2 change of LC3 relative area in presence of NH_4_Cl. Green and orange dots represent known and novel hits respectively. Source data

Autophagy requires the conjugation of the cytosolic LC3 protein (LC3-I) with the lipid phosphatidylethanolamine to form LC3-II on the surface of autophagosomes^25^. The change from a dispersed cytosolic presence of LC3-I to the formation of vesicular structures rich in LC3-II can be exploited in confocal microscopy to visualize autophagosomes. We developed a zebrafish model in which we tagged the fluorescent reporter protein ZsGreen in frame to the 5′ end of *map1lc3*. To drive the expression of the reporter gene in skeletal muscle, we used the skeletal muscle-specific promoter of *actc1b* and generated *Tg(actc1b:ZsGreen-map1lc3;cryaa:TdTomato)* zebrafish, hereafter named actc1b:ZsGreen-LC3 (Fig. 1a,b and Extended Data Fig. 1a). Next, we set up an image acquisition and analysis pipeline that detects LC3-positive puncta for in vivo quantification of autophagosomes (Fig. 1c and Extended Data Fig. 1b). Profiling of zebrafish larvae showed that the number of autophagosomes increased from 3 to 6 days post fertilization (dpf) (Fig. 1c,d). Cotreatment with the lysosomotropic agent ammonium chloride (NH_4_Cl), an inhibitor of autophagosome degradation, increased the accumulation of LC3 puncta compared to baseline from 4 dpf, demonstrating a bona fide increase in autophagic flux (Fig. 1d)^26^. This result is consistent with autophagy activation during the transition of zebrafish larvae from the ‘yolk-feeding’ stage at 3 dpf to a state of limited energy substrate availability due to yolk consumption at 6 dpf (ref. ^27^). To enable the discovery of previously unidentified autophagy modulators in vivo, we implemented a semiautomated multistep protocol as summarized in Fig. 1e and Extended Data Fig. 1c. We opted for an early time window, with analysis at 3 dpf when endogenous autophagic flux is low. Specifically, we distributed larvae at 2 dpf into 96-well microplates. After incubation with the compound of interest, the larvae were immobilized through treatment with an anesthetic before image acquisition as described above. Following the first acquisition, the anesthetic was washed out and the larvae were treated with NH_4_Cl before a second round of imaging was performed.

For validation, larvae were treated with rapamycin, an mTOR inhibitor with well-established effects on autophagy induction in mammals. mTOR activity was abolished after 20 h of treatment of wild-type larvae, as demonstrated by S6 protein phosphorylation (Extended Data Fig. 1d,e). Accordingly, treatment of actc1b:ZsGreen-LC3 larvae for 16 h enhanced autophagic flux in the absence of NH_4_Cl, and the same effect was observed following 4 h of incubation with NH_4_Cl (Fig. 1f,g). Next, we tested the evolutionary conservation of autophagy modulation in zebrafish by performing a pilot screen using a commercial collection of 240 synthetic and natural pharmacophores. Among the seven hits that were identified, three small molecules—ceramide, calpeptin and retinoic acid—have been previously reported to induce autophagy in mammalian cells (Extended Data Fig. 1f and Supplementary Table 1)^28–30^.

These results validate zebrafish as a model for quantifying autophagy levels in vivo, while the semiautomatic analysis pipeline provides a rapid workflow for the discovery of autophagy inducers.

### Carvacrol and thymol are natural autophagy activators

**Fig. 2:**
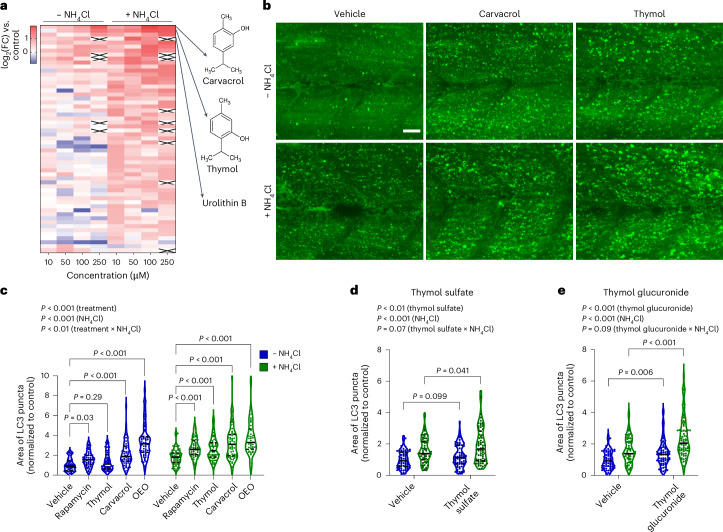
Whole-organism screening identifies thymol and carvacrol as activators of autophagy. **a**, Heat map of autophagy induction in response to bioactives tested at four ascending doses with and without treatment with NH_4_Cl in zebrafish larvae at 3 dpf. The colors represent the relative area of LC3 puncta normalized to the vehicle control, ranging from low (blue) to high (red) activation. White crossed cells represent data excluded due to treatment toxicity (3 dpf; *n* = 4–21 per condition). FC, fold change. **b**, Representative images of ZsGreen-LC3 zebrafish larvae treated for 16 h with the indicated molecules, either alone or in combination with NH_4_Cl. Scale bar, 20 µm. **c**–**e**, Quantification of autophagic flux in transgenic zebrafish larvae treated with 1 µM rapamycin, 50 µM thymol, 50 µM carvacrol and 50 µM oregano essential oil (OEO) (3 dpf; − NH_4_Cl: vehicle, *n* = 59; rapamycin, *n* = 61; thymol, *n* = 61; carvacrol, *n* = 60; OEO, *n* = 60; + NH_4_Cl: vehicle, *n* = 58; rapamycin, *n* = 61; thymol, *n* = 60; carvacrol, *n* = 60; OEO, *n* = 42) (**c**); 50 µM thymol sulfate (3 dpf; vehicle − NH_4_Cl, *n* = 68; vehicle + NH_4_Cl, *n* = 70; thymol sulfate − NH_4_Cl, *n* = 67; thymol sulfate + NH_4_Cl, *n* = 68) (**d**); and 50 µM thymol glucuronide (3 dpf; vehicle − NH_4_Cl, *n* = 70; vehicle + NH_4_Cl, *n* = 73; thymol glucuronide − NH_4_Cl, *n* = 65; thymol glucuronide + NH_4_Cl, *n* = 70) (**e**). Violin plots report the median (solid lines) and quartiles (dotted lines). Data were pooled from three independent experiments and analyzed by two-way ANOVA followed by Sidak multiple comparisons. Source data

**Extended Data Fig. 2:**
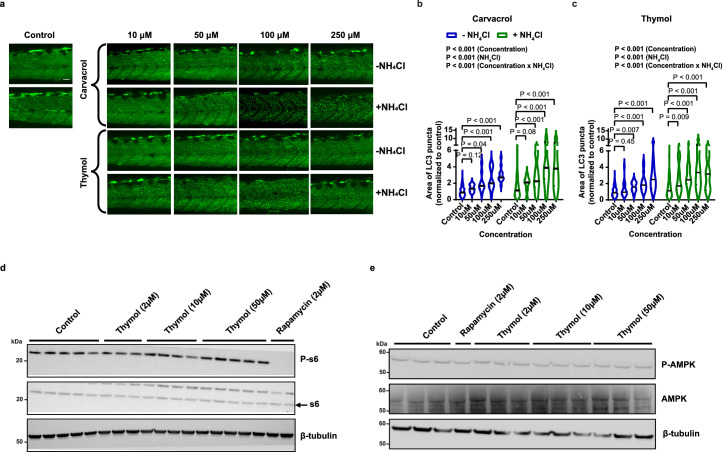
Dose-response effects of thymol and carvacrol on autophagy. **a** Representative images of transgenic zebrafish larvae at 3 dpf treated for 16 h with vehicle (1% DMSO), carvacrol and thymol at the indicated concentrations, followed by treatment with NH_4_Cl for 4 h. Scale bar, 50 µm. **b,c** Quantification of autophagic flux in transgenic zebrafish larvae (3 dpf, n = 17–21). Data are shown as mean ± SEM. Two-way ANOVA followed by Sidak multiple comparisons. **d** Western blot analysis of zebrafish larvae at 3 dpf treated with indicated doses of thymol or 2 µM of rapamycin for phospho-S6 signal activation **e** Western blot analysis of P-AMPK immunoreactivity with same treatments as in **(d)**. Source data

To enrich a screening library for molecules with a favorable safety profile for human translation, we next curated a list of natural bioactive compounds from edible sources and endogenous metabolites, including vitamins, bioactive molecules from herbs and spices, and human serum metabolites (Supplementary Table 2). The molecules were screened at concentrations from 10 to 250 µM and were ranked according to their maximum efficacy (*E*_max_) in the presence of NH_4_Cl (Fig. 2a and Supplementary Table 2). The analysis included high-content imaging of LC3-positive puncta in more than 2,500 larvae in the presence or absence of NH_4_Cl. Carvacrol and thymol, terpenoids abundant in essential oils derived from common thyme (*Thymus vulgaris*) and oregano (*Origanum vulgare*), were the most potent autophagy activators among all the screened bioactive ingredients (Fig. 2a). Images of actc1b:ZsGreen-LC3 larvae demonstrated an accumulation of LC3 puncta at baseline, with a further increase after the addition of NH_4_Cl (Fig. 2b and Extended Data Fig. 2a). Both molecules showed a dose–response relationship, with substantial effects on autophagy starting from 50 µM for carvacrol and 10 µM for thymol (Extended Data Fig. 2b,c). Thymol and carvacrol constitute up to 60% of all phytochemicals in the oil fraction of oregano and thyme^31^. Therefore, we hypothesized that oregano essential oil, a natural product extracted from dry oregano leaves and containing the monoterpenoids carvacrol, *p*-cymene and thymol (Extended Data Table 1), exhibits bioactivity related to autophagy. Consistent with this, we observed that treatment with oregano essential oil induced a robust increase in autophagic flux (Fig. 2c). Notably, thymol, carvacrol and oregano essential oil demonstrated similar efficacy to rapamycin at the doses tested, making them promising candidates for exploring autophagy-dependent health benefits. Considering the structural similarity and comparable efficacy of thymol and carvacrol, we chose thymol as the lead ingredient for subsequent experiments, based on the availability of pharmacokinetic studies in humans^32^. As thymol is metabolized through phase II reactions, we assessed the efficacy of thymol sulfate and thymol glucuronide, the two most prevalent metabolites in human blood after thymol ingestion^32^. Remarkably, both thymol sulfate and thymol glucuronide induced autophagy similarly to free thymol (Fig. 2d,e), indicating that the conjugation of thymol does not interfere with its bioactivity. Thymol did not affect S6 and AMP-activated protein kinase (AMPK) phosphorylation, suggesting that autophagy is not directly activated through these canonical regulatory pathways (Extended Data Fig. 2d,e).

In summary, we identified thymol and carvacrol as potent autophagy inducers that are naturally found in oregano and thyme.

### Thymol is a transient inhibitor of mitochondrial respiration

**Fig. 3:**
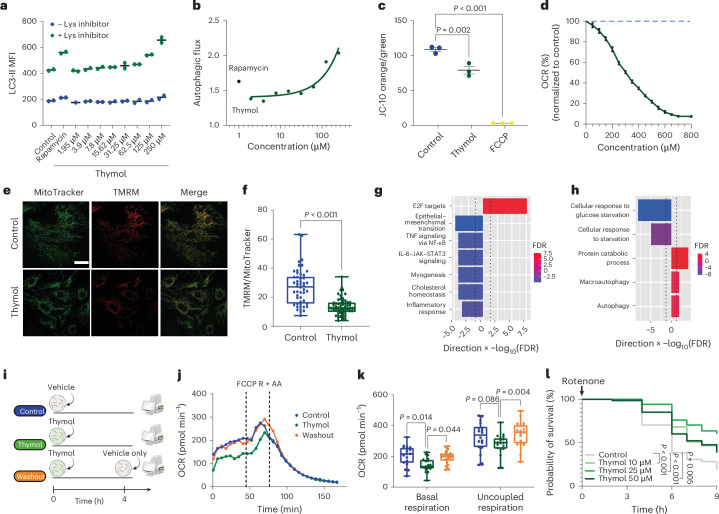
Thymol transiently dampens mitochondrial respiration and protects against mitochondrial toxicity. **a**, LC3 signals measured by flow cytometry in Jurkat cells treated with rapamycin or thymol (blue data points: native; green data points: lysosomal (Lys) inhibitor cotreatment). Each data point represents the mean of a biological replicate (*n* = 2). MFI, mean fluorescence intensity. **b**, Autophagic flux calculated using the LC3-II mean fluorescence intensity: ((+ Lys inhibitor) − (− Lys inhibitor))/(− Lys inhibitor). Data points represent the means of biological replicates (*n* = 2). **c**, MMP in Jurkat cells treated with vehicle (0.1% DMSO), thymol (100 µM) or FCCP (100 nM), measured by flow cytometry as the ratio between the monomeric form (emission wavelength (Em) = 590 nm) and the aggregated form (Em = 525 nm) of JC-10 (*n* = 3 wells per condition). Data are presented as means ± s.e.m. Ordinary one-way ANOVA followed by Sidak multiple comparisons. **d**, ADP-stimulated oxygen consumption rate (OCR) of mitochondria isolated from livers treated with thymol (green solid line). Values were normalized to the vehicle (dashed blue line) and are presented as means ± s.e.m. (*n* = 4). **e**, Microscopy images of MAFs treated with vehicle (0.1% DMSO) or thymol (100 µM) in the presence of MitoTracker (green) and TMRM (red). Scale bar, 50 µm. **f**, Quantification of the TMRM/MitoTracker area to calculate the mitochondrial membrane potential (MMP). Each data point represents one cell (control, *n* = 50; thymol, *n* = 55). Two-tailed Student’s *t* test. **g**, Gene set enrichment analysis in MAFs ranked by level of significance. Dotted lines indicate the significance threshold. FDR, false discovery rate. **h**, Twenty-four significantly enriched pathways related to autophagy and cellular energy metabolism. Experiments (**e**–**h**) were conducted on MAFs with a 24-h exposure to vehicle (0.1% DMSO) or thymol (100 µM) (control, *n* = 6; thymol, *n* = 6). **i**, Setup for OCR analysis. **j**, OCR in zebrafish continuously treated with thymol compared to the washout and control groups (vehicle, 1% DMSO). FCCP stimulates maximal OCR. Rotenone and antimycin A (R + AA) were used to determine nonmitochondrial respiration (3 dpf; control, *n* = 18; thymol, *n* = 21; washout, *n* = 21 pools of larvae). **k**, Basal and maximal OCR calculated as the average of the last three points before the addition of FCCP and R + AA, respectively (3 dpf; control, *n* = 18; thymol, *n* = 20; washout, *n* = 21 pools of larvae). Data were pooled from three independent experiments. Ordinary one-way ANOVA followed by Sidak multiple comparisons. Box plots show the median, 25th and 75th percentiles, and minima and maxima. **l**, Survival analysis of zebrafish larvae treated with thymol or vehicle and exposed to mitochondrial stress through rotenone treatment. Data were pooled from two independent experiments (3 dpf; *n* = 48 per group). Pairwise comparisons to control using a log-rank test. *P* values were adjusted using Bonferroni correction. Flow cytometry gating schemes are shown in Supplementary Fig. 1. Panel **i** created with BioRender.com. Source data

**Extended Data Fig. 3:**
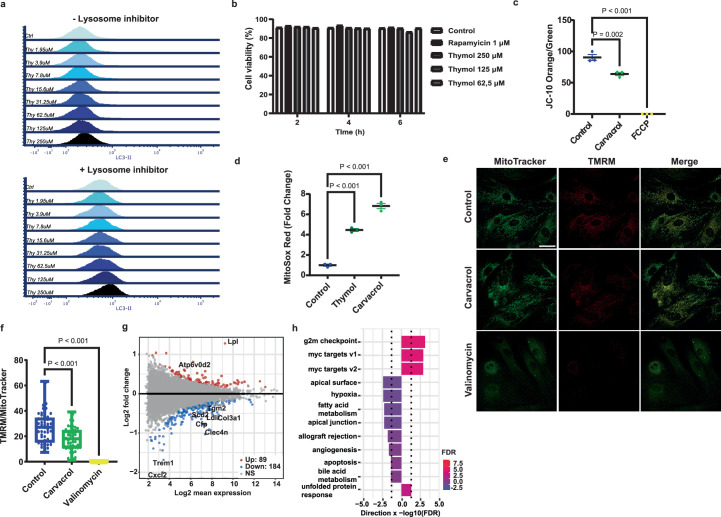
Characterization of thymol and carvacrol bioactivities in mammalian cells. **a** Cytofluorimetric analysis of autophagy flux in Jurkat cells treated with vehicle (ctrl) or thymol for two hours and stained with LC3-II antibody. Representative LC3-II plots show fluorescence intensities in presence (upper panel) or absence (lower panel) of an inhibitor of lysosomal activity. **b** Time-course and dose-response cytofluorimetric analysis of cell viability in Jurkat cells treated with thymol and rapamycin (n = 1). **c** Quantification of mitochondrial membrane potential (MMP) in Jurkat cells treated with control (DMSO 0.1%), carvacrol or FCCP (100 nM) measured as ratio between monomeric (Em = 590 nm) and aggregate forms (Em = 525 nm) of JC-10 (n = 3 wells/condition). **d** Stimulation of ROS generation by thymol (100 µM), carvacrol (100) µM) and FCCP (5 µM) quantified by MitoSox red fluorescence signals (n = 3 wells/condition). Data in c and d are shown as means ± SEM. Ordinary one-way ANOVA followed by Sidak multiple comparisons. **e** Representative confocal microscopy images of Mouse Adult Fibroblasts (MAFs) treated with vehicle (0.1% DMSO), carvacrol (100 µM) and the uncoupler valinomycin (1 µM) in the presence of MitoTracker (green) and TMRM (red) to visualize changes in MMP. Scale bar, 50 µm. **f** Quantification of TMRM/MitoTracker area. Each data point in the boxplots represents one cell (control, n = 50, carvacrol, n = 56, valinomycin n = 50). One-way ANOVA followed by Sidak multiple comparisons. Box plots show median, the 25th and 75th percentiles, and minima and maxima. **g** Scatterplot of QuantSeq 3’ mRNA sequencing analysis in MAFs treated for 24 h with thymol (100 µM) or control (0.1% DMSO). Differentially regulated genes are highlighted in red (up-regulated) and blue (down-regulated); (control, n = 6; thymol, n = 6). **h** Unbiased gene set enrichment analysis in MAFs treated with vehicle and thymol. Gene expression analysis shows remaining significantly enriched gene sets following the top seven presented in Fig. 3g. Dotted lines indicate significance threshold.

We next aimed to determine how thymol induces autophagy and whether the mode of action is conserved across species. Jurkat cells, a human cell line that grows in suspension, can be used to measure autophagic flux through flow cytometry. Treatment with thymol increased the LC3-II signal in the presence of the lysosomal inhibitor starting at 62.5 µM (Fig. 3a,b and Extended Data Fig. 3a). Based on previous reports that thymol intercalates into lipid bilayers, we hypothesized that thymol acts at the level of the mitochondrial membrane to activate autophagy^33–35^. Thymol caused a mild decrease in the MMP, as detected by a fluorometric shift in JC-10 emission from orange, which represents the aggregate form, to green, representing the monomeric form, compared to the chemical uncoupler FCCP (Fig. 3c). Cell viability was not affected even in the presence of 250 µM thymol for up to 6 h, suggesting that autophagy is not a result of cellular toxicity (Extended Data Fig. 3b). To confirm similar bioactivity on mitochondrial membranes, we quantified the MMP in Jurkat cells treated with carvacrol and found a comparable effect to that of thymol (Extended Data Fig. 3c). Consistent with membrane depolarization, treatment with thymol and carvacrol increased mitochondrial superoxide generation, suggesting a mechanism that originates at the mitochondrial membrane (Extended Data Fig. 3d).

Changes in the MMP can affect the respiratory chain and modulate mitochondrial metabolism. Therefore, we measured the oxygen consumption rate (OCR) in mitochondria purified from mouse livers that were treated ex vivo with a range of thymol doses. Consistent with our previous observation that thymol affects the MMP, we found a dose-dependent effect on mitochondrial respiration with a half-maximal inhibitory concentration (IC_50_) of 295.7 ± 6.7 µM in healthy mitochondria (Fig. 3d). Based on these results, we validated the effects through a different method for quantifying the MMP, in which we used primary mouse adult fibroblasts (MAFs) from the same animal model used for OCR quantification. MAFs were treated with thymol for 24 h and then exposed to the fluorescent dye TMRM, a sensitive marker for MMP changes. As expected, TMRM fluorescence was reduced in thymol-treated cells (Fig. 3e), leading to a significant decrease in the TMRM signal compared to that of the control (Student’s *t* test, *P* < 0.001) (Fig. 3f). Carvacrol had similar effects on the MMP (Extended Data Fig. 3e,f).

We next asked whether thymol treatment leads to transcriptional adaptations consistent with changes in cellular energy homeostasis. QuantSeq 3′ mRNA sequencing revealed 273 differentially expressed genes in thymol-treated cells compared to controls (Extended Data Fig. 3g and Supplementary Table 3). Unbiased gene set analysis of cellular hallmarks revealed an anti-inflammatory gene expression signature, with three of the top seven pathways indicating downregulated inflammatory signaling, including tumor necrosis factor (TNF) signaling via nuclear factor-κB (NF-κB) and IL-6–JAK–STAT3 signaling (Fig. 3g). Notably, a gene signature consistent with the activation of an unfolded protein response was upregulated, indicative of a cellular stress response (Extended Data Fig. 3h and Supplementary Table 4). Further, a targeted Gene Ontology analysis of 24 curated biological processes related to cellular energy metabolism and autophagy showed upregulation of pathways linked to macroautophagy, autophagy and protein catabolic processes. In contrast, Gene Ontology terms related to the response to starvation were downregulated (Fig. 3h and Supplementary Table 5).

Depolarization of mitochondria is thought to induce autophagy and mitophagy, thereby initiating the targeted removal and replacement of cellular components, which results in improved resilience to energetic stressors and damage^36^. To be beneficial, this mechanism needs to be transient, without long-term inhibitory effects on cellular respiration, and should be moderate enough to predominantly affect damaged mitochondria. We first tested the kinetics of thymol’s effects on mitochondrial respiration by measuring the OCR in zebrafish larvae. In this setup, we compared a vehicle group to a group of animals that were continuously exposed to thymol and to another group in which thymol treatment was replaced by vehicle treatment 30 min before the analysis (Fig. 3i). Consistent with the in vitro experiments in mammalian cells, thymol reduced basal respiration in larvae, with minor effects on uncoupled respiration, suggesting that thymol does not affect maximal respiratory capacity. Importantly, mitochondrial function was fully rescued within 30 min following the removal of thymol (Fig. 3j,k). We hypothesized that transient inhibition of mitochondrial respiration leads to autophagy, thereby removing toxic materials and improving overall cellular quality. To test whether thymol increases organismal resilience, we treated zebrafish larvae with the mitochondrial complex I inhibitor rotenone, a toxin whose effects have been previously shown to be counteracted by the activation of autophagy and mitophagy^37^. Pretreatment of larvae with thymol for 16 h at three different doses reduced rotenone-induced toxicity, indicating that thymol primes cells for improved resilience to mitochondrial damage (Fig. 3l).

In summary, these results show that the effects of thymol on mitochondrial membrane integrity and respiratory function are transient, reversible, and confer protection at both the cellular and organismal levels.

### Thymol induces mitophagy in cells and mice

**Fig. 4:**
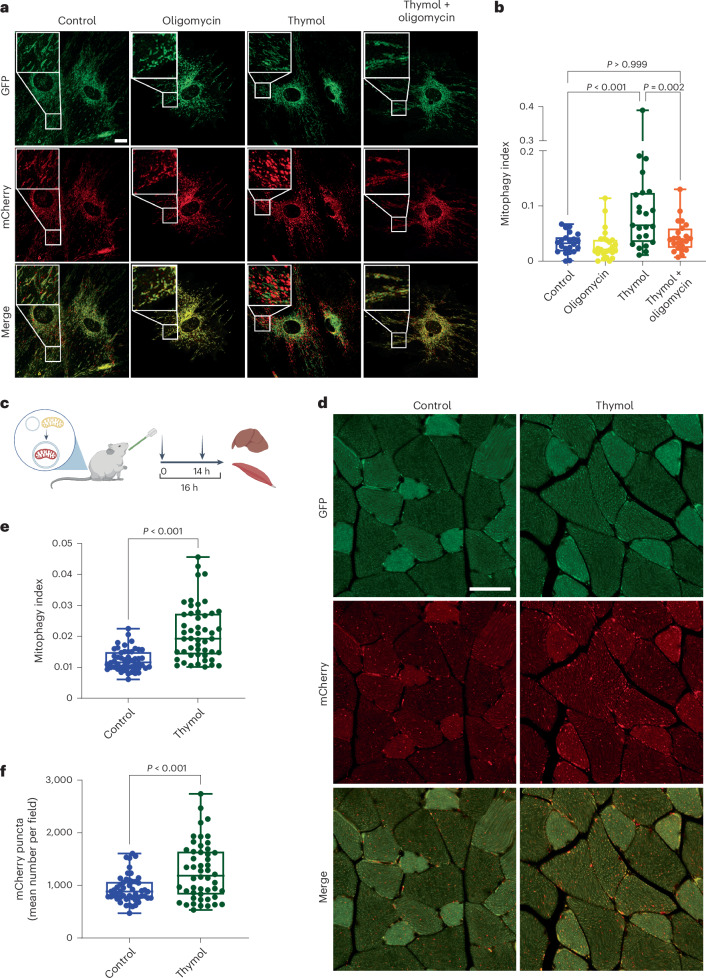
Thymol induces mitophagy in cells and mice. **a**, Representative confocal microscopy images of mito-QC MAFs treated with vehicle (0.1% DMSO), oligomycin (1 nM), thymol (100 µM) or thymol (100 µM) + oligomycin (1 nM) for 24 h. Scale bar, 20 µm. **b**, Quantification of mitophagy activation as the mitophagy index, which represents the relative mitolysosomal area in mito-QC MAFs. Each data point in the box plots represents one cell (control, *n* = 22; oligomycin, *n* = 25; thymol, *n* = 23; thymol + oligomycin, *n* = 23). Ordinary one-way ANOVA followed by Dunnett multiple comparisons. **c**, Study scheme for the acute exposure of transgenic mito-QC reporter mice to vehicle or thymol. Mice received two doses of thymol by oral gavage at a dose of 20 mg per kg body weight, or vehicle, 16 and 2 h before liver and muscle tissue collection (10 weeks old, *n* = 5 per group). **d**, Representative confocal microscopy images of gastrocnemius skeletal muscle 2 h after the second treatment. Scale bar, 50 µm. **e**, Quantification of the mitophagy index as the relative mitolysosomal area. **f**, Number of mCherry-positive mitophagy foci. Ten fields were analyzed for each mouse, with each data point in the box plots in **e** and **f** representing the value from an individual microscopic field (control, *n* = 50; thymol, *n* = 50). Two-tailed Student’s *t* test. Box plots show the median, 25th and 75th percentiles, and minima and maxima. Panel **c** created with BioRender.com. Source data

**Extended Data Fig. 4:**
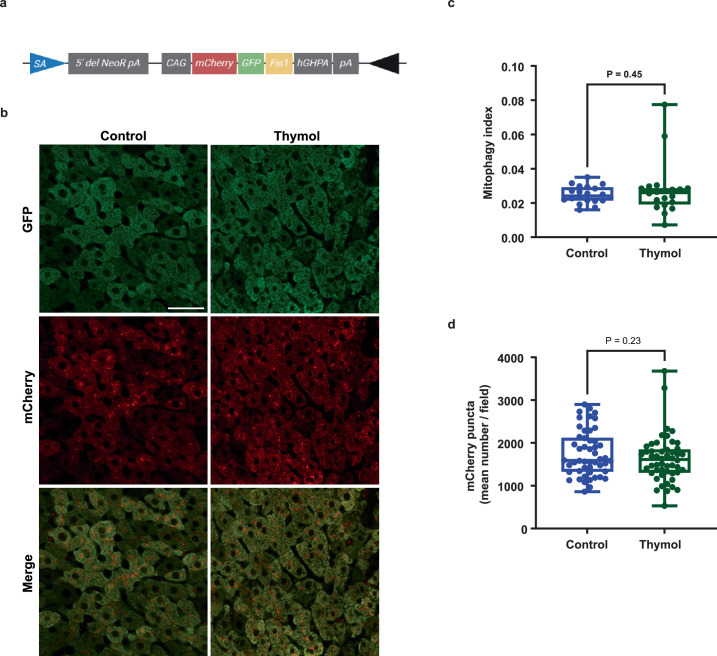
Lack of effects of thymol on mitophagy activation in liver tissue of mitoQC mice. **a** Transgenic construct to quantify mitophagy in mito-QC mice. **b** Representative confocal microscopy images of liver tissue 2 h hours after the second treatment. Note mosaic expression of the transgenic reporter proteins in hepatic tissue. Scale bar, 50 µm. **c** Quantification of mitophagy as mitophagy index. **d** Number of mCherry-positive mitophagy foci. For each mouse, ten fields were analyzed, with each data point in the boxplots c and d representing the value from an individual microscopy field. (control, n = 50; thymol, n = 50). Two-tailed Student’s t test. Box plots show median, the 25th and 75th percentiles, and minima and maxima. Panel **a** created with BioRender.com.

The activation of autophagy and the transient inhibition of mitochondrial respiration suggest that both mechanisms induced by thymol are functionally connected. To test whether thymol’s effect of lowering of the MMP triggers the activation of mitophagy in addition to macroautophagy, we obtained MAFs derived from mito-QC reporter mice that carry a tandem mCherry-GFP tag fused to the mitochondrial targeting sequence of the outer mitochondrial membrane protein FIS1 (encoded by *Fis1*)^38^ (Extended Data Fig. 4a). Fibroblasts were derived from male mito-QC mice. Of note, all mice used in the study, including those receiving in vivo treatments, were male. When mitophagy is activated in these mice, mitochondria tagged by the reporter construct are delivered to lysosomes, where GFP fluorescence is quenched by the acidic environment of the lumen, resulting in red foci of mitochondria due to the remaining fluorescence emission of the mCherry signal. Treatment with thymol for 24 h led to a strong activation of mitophagy, measured by the appearance of red fluorescent puncta and quantified as the mitophagy index, defined as the ratio of mCherry-positive mitolysosomes to the total number of mCherry-GFP-positive mitochondria (Fig. 4a,b). In contrast, cotreatment with oligomycin, an inhibitor of mitochondrial ATP synthase that blocks proton efflux and prevents mitochondrial depolarization, attenuated the effect of thymol on mitophagy (Fig. 4a,b). Thus, thymol-induced mitochondrial depolarization is necessary to initiate the mitophagy process. Next, we turned to an in vivo mammalian model and performed oral gavage with two administrations of thymol at 20 mg kg^−1^ per dose using mito-QC reporter mice (Fig. 4c). Gastrocnemius muscle biopsies and liver samples were collected 2 h after the last treatment, and histological sections were imaged to assess mitophagy levels (Fig. 4d and Extended Data Fig. 4b). Oral thymol treatment resulted in a striking induction of mitophagy in skeletal muscle, as demonstrated by increases in the mitophagy index and the number of mCherry-positive mitochondria (Fig. 4e,f). In contrast, mitophagy activation in liver samples was similar between the thymol and vehicle groups, potentially limited by the timing of sampling relative to the pharmacodynamics of thymol, or due to the mosaic expression of the transgene as reported previously (Extended Data Fig. 4c,d)^38,39^.

Taken together, our results show that mitophagy activation by oregano terpenoids occurs both in vitro and in vivo and is triggered by a mechanism that requires mild and transient mitochondrial depolarization.

### Thymol protects against liver fat accumulation in mice

**Fig. 5:**
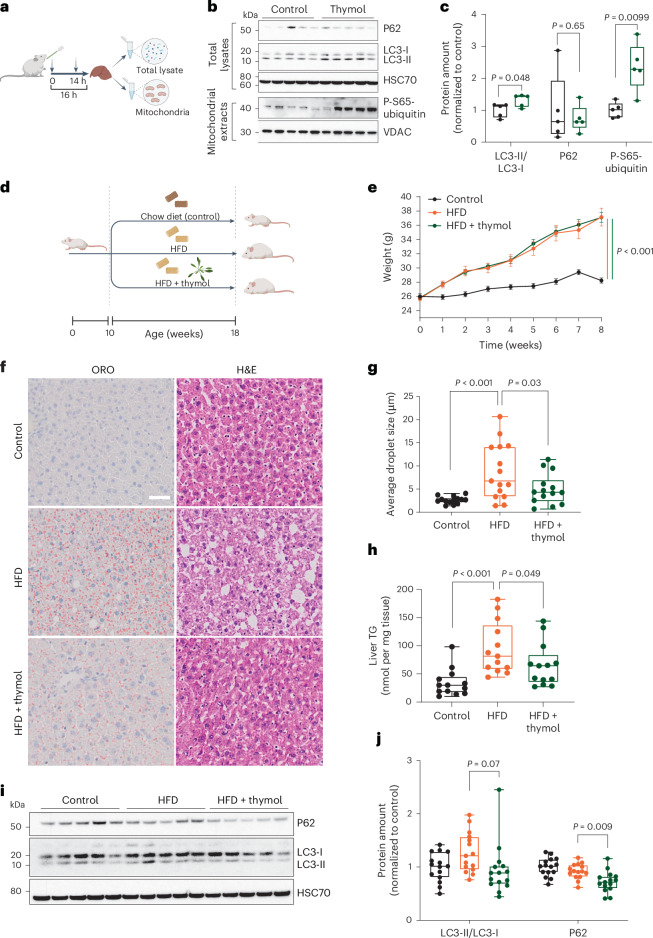
Thymol prevents liver fat accumulation in mice fed a HFD. **a**, Schematic overview of acute intervention with thymol in C57BL/6 mice. Mice received either thymol 20 mg kg^−1^ or vehicle by two gavages 16 and 2 h before they were killed, and proteins or mitochondria were isolated from liver tissues after animal killing (10 weeks old, *n* = 5 per group). **b**, Western blot analysis of total lysates and mitochondrial extracts from liver of mice treated with thymol or vehicle using the indicated antibodies. **c**, Densitometric quantification of the western blots. One-tailed Student’s *t* test. **d**, Schematic overview of chronic intervention study in wild-type mice. Ten-week-old mice were fed standard chow (control) or a HFD. Thymol or vehicle was administered per os to mice fed a HFD 5 days per week for 8 weeks. The control group was treated with vehicle only. **e**, Body weight changes throughout the intervention. Data are presented as means ± s.e.m. with pairwise comparisons at the end of the treatment (*n* = 15 mice per group). Two-tailed Student’s *t* test. **f**, Representative histological images of liver tissue stained with Oil Red O (ORO) and hematoxylin and eosin (H&E). Scale bar, 100 µm. **g**, Quantification of mean lipid droplet size from ORO staining. Each data point represents the value quantified from one mouse (control + vehicle, *n* = 14; HFD + vehicle, *n* = 15; HFD + thymol, *n* = 14). **h**, Liver triglyceride (TG) content (*n* = 13 per group). **i**, Western blot analysis of liver tissues using the indicated antibodies for the quantification of LC3-II/LC3-I and P62 protein contents. **j**, Densitometric quantification of the western blots (*n* = 15 per group). Box plots in **c**, **g**, **h** and **j** show the median, 25th and 75th percentiles, and minima and maxima. Ordinary one-way ANOVA followed by Sidak multiple comparisons. Panels **a** and **d** created with BioRender.com. Source data

Autophagy enables the degradation of triglyceride-rich lipid droplets in hepatocytes, protecting against aberrant liver fat accumulation, a common phenotype in aging that is compounded by calorie-dense but nutrient-poor Western diets^40^. As thymol inhibits the respiratory capacity of liver mitochondria at elevated doses (Fig. 3d), we tested its effects on hepatic macroautophagy and mitophagy by western blot analysis in wild-type mice. Total tissue lysate and mitochondria were purified from liver tissue to determine markers of autophagy and mitophagy in response to oral treatment (Fig. 5a). Thymol increased the autophagic flux, measured as the LC3-II/LC3-I protein ratio, in conjunction with hyperphosphorylation of mitochondrial ubiquitin at the Ser-65 residue, suggesting mitophagy activation through canonical PINK-1/Parkin signaling (Fig. 5b,c). In contrast, the levels of the autophagy target P62 remained unchanged, potentially due to the variable timing with which these markers respond during different stages of the autophagic flux^41^.

**Extended Data Fig. 5:**
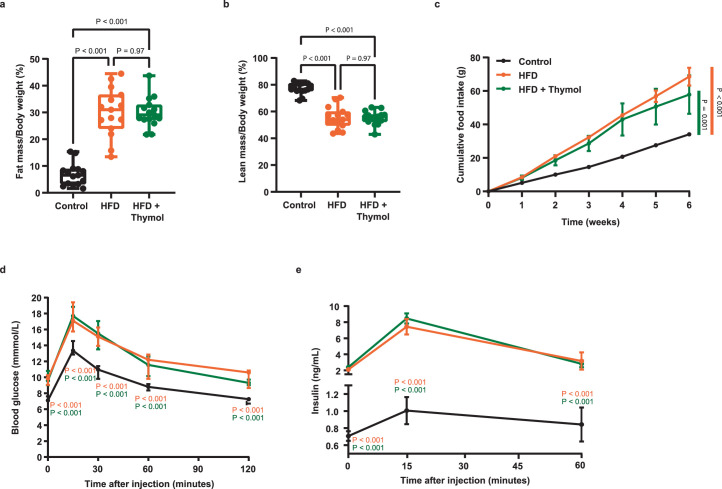
Effects of high fat diet and thymol treatment on body mass and insulin resistance in mice. **a,b** Whole body composition analysis in mice by multi-echo magnetic resonance imaging (MRI). Percentages of fat mass **(a)** and lean mass **(b)** were measured at the end of the treatment. Boxplots show media, the 25th and 75th percentiles, and minima and maxima (n = 15). One-way ANOVA followed by Sidak multiple comparisons. **c** Cumulative food intake of mice fed with control and high fat diet and treated with thymol or vehicle only. **d** Oral glucose tolerance test (OGTT) performed after 7 weeks of intervention. Blood glucose levels were measured after glucose challenge at the indicated timepoints. **e** Blood insulin concentrations after glucose challenge at the indicated timepoints. Data show mean ± SEM (n = 15) and are analyzed by one-way ANOVA followed by Sidak multiple comparisons. P values in **d** and **e** are color coded: orange for high fat diet versus control and green for high fat diet and thymol versus control.

Following this acute study, we set out to test whether thymol prevents the accumulation of excess liver fat in chronic conditions. Groups of mice were fed a high-fat diet (HFD) and supplemented with 20 mg kg^−1^ of thymol or a vehicle control by gavage for 5 days per week over 8 weeks. A group of mice was fed a standard chow diet and received vehicle treatment by gavage to serve as a reference (Fig. 5d). At the end of the treatment, body weight was higher in mice fed a HFD than in their littermates that were fed a standard diet (Fig. 5e). Thymol had no effects on weight gain (Fig. 5e), body composition (Extended Data Fig. 5a,b) or food intake (Extended Data Fig. 5c). Blood glucose and insulin responses to an oral glucose tolerance test (OGTT) were similar between the thymol-treated and control HFD-fed groups (Extended Data Fig. 5d,e). In contrast, liver histological analysis revealed robust protection against steatosis and preserved tissue morphology in thymol-treated mice compared to the vehicle-treated controls (Fig. 5f). Oil Red O (ORO) analysis showed that the amount of lipid droplets decreased in the livers of animals treated with thymol, along with a reduction in tissue triglyceride content (Fig. 5g,h). Consistent with an enhancement of autophagy, we observed lower levels of P62, an autophagy cargo adaptor and substrate that becomes depleted in response to increased autophagic flux^10^ (Fig. 5i,j). No differences between groups were observed for the LC3-II/LC3-I ratio, suggesting that autophagic flux stabilizes over the treatment period and leads to the degradation of autophagosomes and their cargo, including P62, without further changes in the LC3-II/LC3-I ratio.

Taken together, oral thymol intake acutely activates liver autophagy and mitophagy, preventing excess liver fat accumulation during a chronic HFD challenge, independent of changes in body weight.

### Thymol improves healthspan in *C. elegans*

**Fig. 6:**
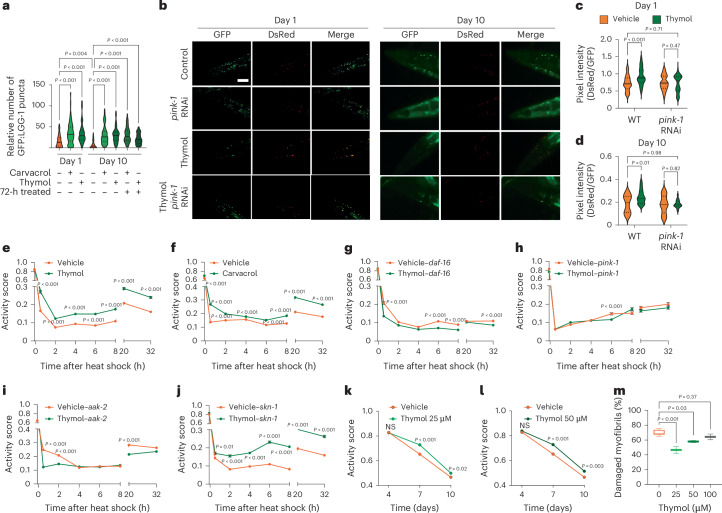
Thymol increases *pink-1-*dependent stress resistance, improves motility and reduces age-related myofibril damage in *C. elegans.* **a**, Mean number of GFP-positive puncta per individual in treated compared to control animals (day 1: control, *n* = 149; carvacrol, *n* = 193; thymol, *n* = 132; day 10: control, *n* = 109; carvacrol, *n* = 138; thymol, *n* = 114; 72-h pretreated: carvacrol, *n* = 79; thymol *n* = 61). Ordinary one-way ANOVA followed by Sidak multiple comparisons. **b**, Epifluorescence microscopy images of *C. elegans* expressing an mtRosella biosensor in body wall muscle cells to visualize mitophagy in response to control (0.1% DMSO) and thymol (25 µM) treatment in young (day 1) and old (day 10) wild-type and *pink-1* (RNAi)-treated animals. Scale bar, 8 µm. **c**,**d**, Mitophagy quantified on day 1 (**c**) (wild-type (WT): vehicle, *n* = 80; thymol, *n* = 63; *pink-1* RNAi: vehicle, *n* = 52; thymol, *n* = 66) and day 10 (**d**) (WT: vehicle, *n* = 15; thymol, *n* = 16; *pink-1* RNAi: vehicle, *n* = 16; thymol, *n* = 18) as the ratio of DsRed to GFP fluorescence intensity. Violin plots show the median (solid lines) and quartiles (dashed lines). Two-way ANOVA followed by Sidak multiple comparisons. **e**,**f**, Activity score (arbitrary units) in response to 4 h of thermal shock at 37 °C. Before heat exposure, worms were treated with thymol (vehicle, *n* = 2,319; thymol, *n* = 1,728) (**e**) or carvacrol (vehicle, *n* = 2,266; carvacrol, *n* = 1,980) (**f**) for 72 h at 50 μM from days 1 to 3 of adulthood. **g**–**j**, Activity quantification upon genetic loss-of-function of *daf-16* (vehicle, *n* = 1,677; thymol, *n* = 1,791) (**g**), *pink-1* (vehicle, *n* = 1,275; thymol *n* = 1,295) (**h**), *aak-2* (vehicle, *n* = 2,500; thymol, *n* = 2,234) (**i**) or *skn-1* (vehicle, *n* = 1,800; thymol *n* = 1,497) (**j**) in response to 50 μM thymol following the same conditions as in **e**. **k**,**l**, Activity score on days 4, 7 and 10 of adulthood in worms exposed to thymol at 25 μM (vehicle, *n* = 1,068; thymol *n* = 1,029) (**k**) and 50 μM (vehicle, *n* = 1,068; thymol, *n* = 1,044) (**l**) or vehicle (1% DMSO) from the egg stage to adulthood. Ordinary one-way ANOVA followed by Dunnett multiple comparisons. In **e**–**l**, data were pooled from three independent experiments and are presented as means ± s.e.m. **m**, Percentage of damaged myofibrils in 10-day-old *C. elegans* in response to lifelong treatment with thymol. Box plots show the median, 25th and 75th percentiles, and minima and maxima. Ordinary one-way ANOVA followed by Sidak multiple comparisons. Data were pooled from two independent experiments, with 200 muscle fibers analyzed for each condition. NS, no significance. Source data

**Extended Data Fig. 6:**
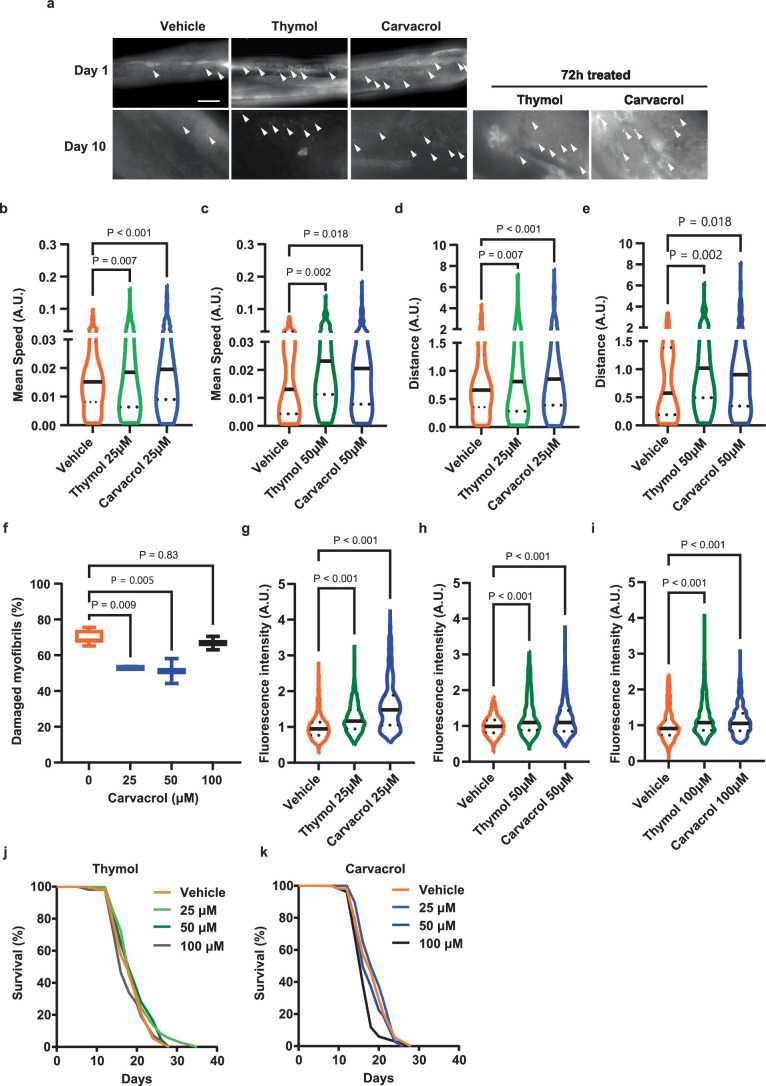
Effects of thymol and carvacrol on healthspan and lifespan of *C. elegans.* **a** Fluorescence microscopy images of transgenic GFP::LGG-1 reporter *C. elegans* visualizing the activation of autophagy through quantification of green fluorescent foci in body wall muscle cells. Young (Day 1) and aged (Day 10) worms were treated with thymol and carvacrol at 25 µM compared to vehicle (DMSO, 0.1%). Duration of administration was lifelong treatment (left panels) or 72 h (right panels) prior to imaging. Arrows indicate GFP::LGG-1 positive punctae, indicative of autophagosomes. **b-e** Motility assays in Day 10 *C. elegans* treated with thymol or carvacrol at the indicated doses. Mean speed (**b,c**) and total distance travelled (**d,e**) were quantified to assess age-related decline in motility functions. At least 92 worms were analyzed for each condition**. f** Percentage of damaged myofibrils in Day 10 old *C. elegans* in response to lifelong treatment with thymol at indicated doses. Boxplots show median, the 25^th^ and 75^th^ percentiles, minima and maxima. Ordinary One-way ANOVA followed by Sidak multiple comparisons. Data are pooled from two independent experiments, with at least 200 muscle fibers analyzed for each condition. **g-i** Quantification of damaged myofibrils as function of fluorescence intensity in response to thymol or carvacrol at indicated concentrations. Data are pooled from two independent experiments, with at least 300 muscle fibers analyzed for each condition. Ordinary One-way ANOVA followed by Sidaj multiple comparisons. Box plots show median, the 25th and 75th percentiles, and minima and maxima. Violin plot shows median (solid lines) and quartiles (dashed lines). **j,k** Survival analysis of *C. elegans* exposed to indicated doses of thymol **(j)** and carvacrol **(k)** throughout lifespan. At least 50 worms per condition have been analyzed and compared with vehicle using log rank test.

*Caenorhabditis elegans* is a well-suited model organism for studying epistatic mechanisms that mediate the effects of small-molecule interventions on healthspan^42^. We first aimed to confirm an evolutionarily conserved effect of thymol and carvacrol on autophagy induction. We treated wild-type worms that carry a transgene in which *GFP* is fused to the *C. elegans* ortholog of *MAP1LC3*, leading to the expression of a GFP::LGG-1 reporter. Worms were exposed to thymol or carvacrol at different life stages and for varying durations of exposure. Thymol and carvacrol increased GFP::LGG-1 puncta in young (day 1) and adult worms in response to lifelong treatment (Fig. 6a and Extended Data Fig. 6a). The same effect was observed in aged animals (10 days old) treated throughout their lives or in response to a subchronic duration of 72 h (Fig. 6a and Extended Data Fig. 6a). Notably, autophagy levels in aged *C. elegans* treated with the terpenoids were robust and comparable to those observed in young worms, despite lower baseline autophagy levels (Fig. 6a and Extended Data Fig. 6a). Thus, thymol is effective in overcoming age-related declines in autophagy levels. Based on the ability to induce autophagy in *C. elegans*, we used a transgenic line carrying a mitochondria-targeted Rosella (mtRosella) reporter in the muscle cells of the body wall. This dual-fluorescent reporter coexpresses pH-sensitive GFP (mtGFP) and pH-insensitive DsRed. In this setup, we tested the ability of thymol to activate mitophagy in wild-type worms and *pink-1* mutants lacking the membrane-bound PINK-1 protein, which initiates canonical mitophagy upon membrane depolarization^43^. Thymol robustly increased mitophagy signals in both young and aged worms (Fig. 6b–d). The reversal of age-related autophagy decline was absent in worms lacking functional PINK-1 signaling, demonstrating that thymol signals through canonical mitophagy activation (Fig. 6c,d).

Exposing cells to nonphysiological temperatures induces the expression of heat shock proteins, a group of chaperones that protect cellular components by forming protein aggregates^44^. We hypothesized that thymol increases resilience to heat stress through the turnover of protein aggregates and damaged mitochondria, thereby reinstating proteostasis that is perturbed by high temperatures. Exposure of *C. elegans* to a temperature of 37 °C for 4 h sharply reduced their activity score, a measure that assesses animal motility as a readout of recovery from the heat exposure (Fig. 6e–j). Thymol or carvacrol treatments 72 h before the heat shock robustly improved thermotolerance, measured as the percentage of active animals during a time course of 32 h after the thermal shock (Fig. 6e,f). We repeated the same experiment in worms with genetic deletions of *daf-16*, which is critical for autophagy signaling, and *pink-1*, the gatekeeper of canonical mitophagy signaling, to test dependency on these pathways^45^. Thymol did not protect worms deficient in *daf-16* and *pink-1*, indicating that these genes are required for the benefits of thymol (Fig. 6g,h). *C. elegans* deficient in *aak-2*, the ortholog of genes encoding proteins of the AMPK complex in vertebrates, also interfered with thymol-dependent motility benefits, consistent with the upstream regulatory role of AMPK in the induction of autophagy and mitophagy^46^ (Fig. 6i). In contrast, the effects of thymol did not depend on *skn-1*, which encodes a transcription factor with conserved functions in oxidative stress responses to NRF2 in mammals^47^, indicating that the protective effects do not rely on reactive oxygen species signaling (Fig. 6j).

Next, we assessed motility as a surrogate for improved healthspan during lifelong treatments in microfluidic chambers. Activity scores improved in worms exposed to thymol at concentrations of 25 and 50 µM from 7 to 10 days of adulthood (Fig. 6k,l). Similar benefits regarding age-related motility were also observed when using conventional microplates, with thymol and carvacrol at concentrations of 25 and 50 µM consistently enhancing velocity and total traveled distance following treatments from the egg stage to 10 days of adulthood (Extended Data Fig. 6b–e).

Lastly, we asked whether the improved motility outcomes were also reflected in preserved anatomical structures. To this end, we used a strain that expresses MYO-3 tagged with GFP in body wall muscles and quantified the damage to myofibrils, which consist of the contractile proteins required for efficient movement. During aging, myofibers exhibit severe damage and decreased MYO-3 expression^48^. Thymol and carvacrol treatments preserved myofiber structure at 25 and 50 µM, while higher doses did not reduce damage (Fig. 6m and Extended Data Fig. 6f), suggesting that higher doses may lack benefits under chronic exposure. Moreover, both thymol and carvacrol increased the MYO-3::GFP fluorescence intensity at all tested doses, indicating healthier myofibrils (Extended Data Fig. 6g–i). We did not observe an effect on overall lifespan, potentially due to antagonistic effects under chronic exposure, among other reasons (Extended Data Fig. 6j,k).

In summary, thymol improves age-related motility and slows skeletal muscle decline by enhancing cellular resilience to aging and stress through the activation of autophagy and mitophagy.

### Thymol delays epigenetic aging and physical decline in mice

**Fig. 7:**
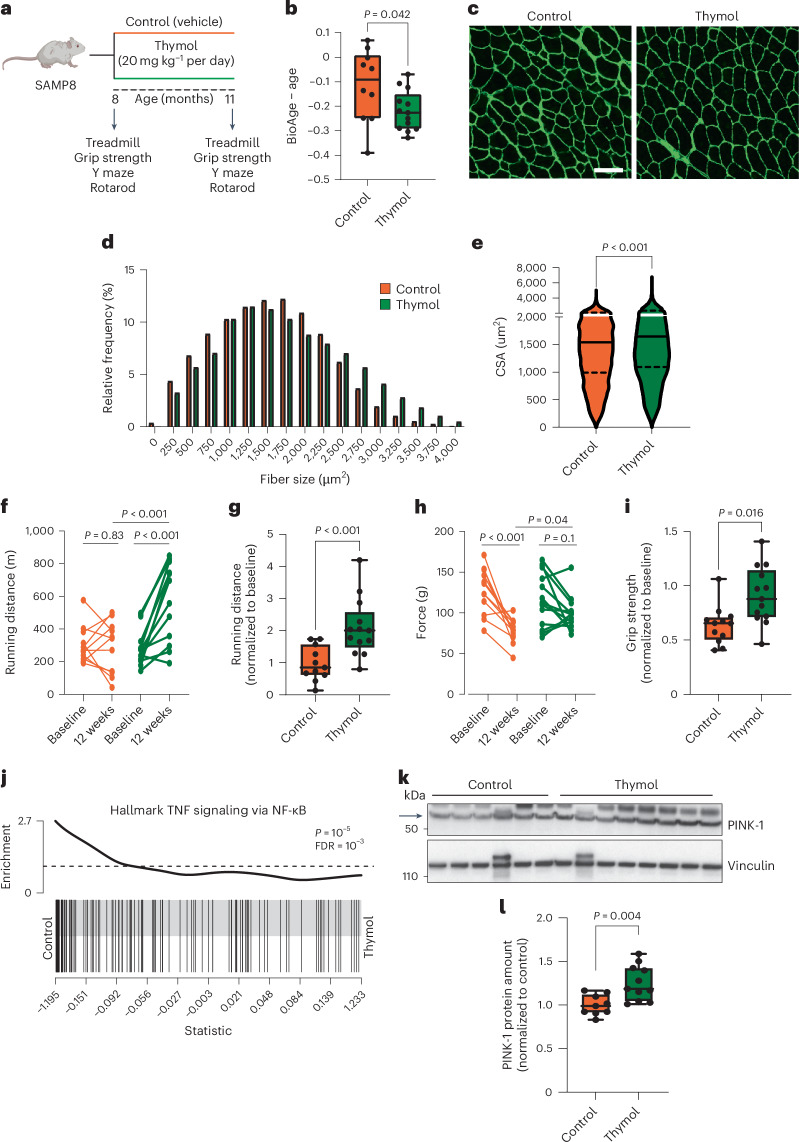
Thymol delays biological aging, prevents sarcopenia and improves performance in mice with accelerated aging. **a**, Schematic overview of chronic interventions with thymol in SAMP8 mice (*n* = 15 per group). **b**, Biological age measured in skeletal muscle through the quantification of epigenetic clock-specific methylation sites, expressed as biological age minus chronological age (BioAge − age) in SAMP8 mice (control, *n* = 10; thymol, *n* = 13). **c**, Representative immunofluorescence images of the tibialis anterior muscle stained with laminin. Scale bar, 20 µm. **d**, Frequency distribution of skeletal muscle fibers according to their cross-sectional area. **e**, Mean cross-sectional area (CSA) of tibialis muscle fibers. The violin plot shows the median (solid lines) and quartiles (dashed lines). **f**, Treadmill analysis at baseline and after 12 weeks of treatment (control, *n* = 11 mice; thymol, *n* = 13 mice). **g**, Treadmill analysis results expressed as the ratio of running distance at the end of the study to that at baseline (control, *n* = 11; thymol, *n* = 13). **h**, Grip strength measured at baseline and after 12 weeks of treatment (control, *n* = 12 mice; thymol, *n* = 13 mice). **i**, Grip strength expressed as the ratio of force between the end of the study and baseline (control, *n* = 12 mice; thymol, *n* = 13 mice). **j**, Gene set enrichment barcode plot for the TNF signaling via NF-κB gene set from the 3′ QuantSeq analysis of the gastrocnemius. **k**, Western blot analysis of skeletal muscle from mice treated with thymol or vehicle using the indicated antibodies. **l**, Densitometric quantification of the western blots. Each data point represents the densitometric value obtained from individual muscle lysates (control, *n* = 9; thymol, *n* = 10). Data points in the before–after plots in **f** and **h** represent values from each mouse at baseline and the end of the study (12 weeks). Box plots show the median, 25th and 75th percentiles, and minima and maxima. One-tailed Student’s *t* test. Panel **a** created with BioRender.com. Source data

The effects of thymol on autophagy across species and tissue types suggest broad benefits that collectively support healthy aging. To this end, we aimed to translate these findings to a mammalian model of progressive aging by studying SAMP8 (senescence-accelerated mouse prone 8) mice. This model is particularly suited for studying the aging of the brain and physical performance, two domains that have a major role in age-related disabilities in humans. SAMP8 mice are an inbred strain selected from a genetically heterogeneous population of mice. Specifically, these mice naturally develop early signs of aging, such as reduced locomotor performance, sarcopenia, and cognitive decline linked to an accumulation of protein aggregates and a loss of mitochondrial respiratory function. Therefore, they are considered a relevant model for human aging^49,50^. We administered thymol at a dose of 20 mg kg^−1^ per day or vehicle by oral gavage for 5 days per week over 12 weeks starting at 8 months of age (Fig. 7a). First, we analyzed DNA methylation patterns that predict age-adjusted health status. These methylation-based epigenetic clocks are emerging as relevant biomarkers of aging in mammals and are used to detect the beneficial effects of interventions designed to extend healthspan^51^. Notably, the skeletal muscle tissue of mice supplemented with thymol showed a reduction in epigenetic age, as assessed using a pan-tissue epigenetic clock^52^, of approximately 20% compared to the control group (Student’s *t* test, *P* = 0.033) (Fig. 7b).

**Extended Data Fig. 7:**
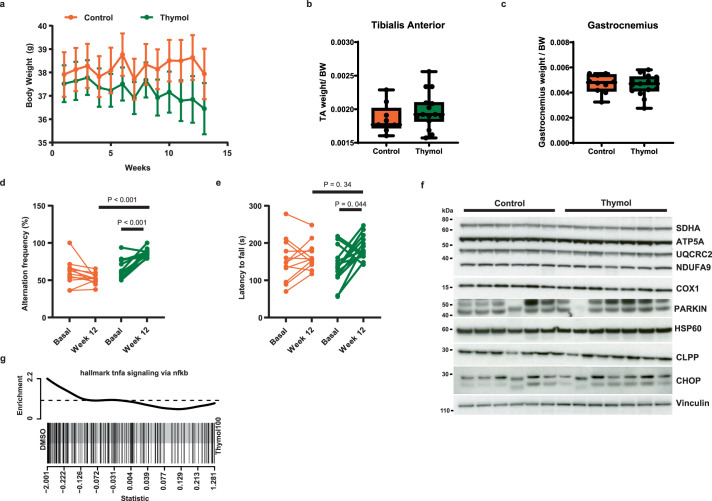
Effects of thymol on age-related outcomes in SAMP8 mice. **a** Weekly body weight analysis of mice from start to end of treatment (n = 15 mice/group). Mice that died were excluded from subsequent analyses **b, c** Average weights of tibialis anterior **(b)** and gastrocnemius **(c)** muscles (Control, n = 10; thymol, n = 13) **d** Spatial memory performance measured by Y maze at the baseline and after 12 weeks of treatment (Control, n = 11; thymol, n = 13). **e** Motor performance measured as latency to fall in a rotarod test (Control, n = 11; thymol, n = 13). Datapoints in the before-after plots in panels **d** and **e** represent values from each mouse at the baseline and at the end of the study (12 weeks). **f** Western blot analysis of total lysate extracts of skeletal muscle from mice treated with thymol or vehicle using the indicated antibodies. **g** Gene enrichment for tnfα signaling via nfkb pathway from 3’ QuantSeq analysis of MAFs shown as barcode plot. Box plots show median, the 25^th^ and 75^th^ percentiles, minima and maxima. Two-tailed Student’s *t* test. Source data

Similar to findings in mice fed a HFD, thymol treatment did not affect body weight (Extended Data Fig. 7a). Histological analysis of the tibialis anterior muscle showed larger myofibers in mice treated with thymol compared to controls (Fig. 7c). The frequency distribution of the muscle fiber area further showed a shift toward larger myofibers compared to untreated mice (Fig. 7d), resulting in an approximately 13% increase in the cross-sectional fiber area (Student’s *t* test, *P* < 0.001) (Fig. 7e). Endurance was strikingly higher in the thymol-treated group than in the control group (Fig. 7f). Notably, average performance increased twofold from baseline in treated mice, whereas it remained stable in the controls, suggesting an improvement in performance induced by thymol, even in a life stage characterized by a decline in mobility (Fig. 7g). Grip strength was preserved throughout the study in the treatment group, showing a robust preventive effect against the decline of muscle strength compared to control animals (Fig. 7h). Numerically, strength decreased by 36% from baseline in the vehicle-treated controls but remained stable from baseline in the thymol-treated group (Fig. 7i). The weights of the tibialis anterior and gastrocnemius muscles did not show differences between the groups (Extended Data Fig. 7b,c). Furthermore, motor coordination and working memory capacity, measured by the rotarod test and the continuous Y maze task, improved in the group of mice treated with thymol (Extended Data Fig. 7d,e). Despite the remarkable phenotypic improvements, the levels of mitochondrial respiratory chain proteins and protein markers of the mitochondrial unfolded protein response remained unchanged, suggesting a relatively selective effect on mitophagy independent of major changes in the total mitochondrial mass (Extended Data Fig. 7f). Gene expression changes were moderate, with only one gene set related to anti-inflammatory responses showing significant enrichment (Fig. 7j and Supplementary Table 6). Of note, the gene set—hallmark TNF signaling via NF-κB—was similar to that found in MAFs in response to thymol treatment (Extended Data Fig. 7g). Western blot analysis of gastrocnemius tissue biopsies showed an increased amount of PINK-1 protein, indicating robust activation of mitophagy through canonical PINK-1 signaling (Fig. 7k,l).

Together, these data indicate that thymol treatment delays the age-related decline in muscle performance and lowers epigenetic age in SAMP8 mice.

## Discussion

Autophagy has emerged as a principal mechanism that maintains cellular functions during aging^53^. Here, we describe thymol and carvacrol, two isomeric phenolic monoterpenes derived from thyme and oregano, as inducers of autophagy and mitophagy both in vitro and in vivo, with conserved effects across species. Thymol and carvacrol are hydrophobic molecules with bactericidal properties due to their ability to intercalate into bacterial membranes, and they are thought to provide antipathogenic defenses^33–35^. Mitochondria evolved from ancestral proteobacteria through an endosymbiotic process, giving rise to the bioenergetic properties of aerobic eukaryotes^54^. Autophagy induction by thymol in our eukaryotic models is consistent with an effect on mitochondrial membranes that resembles bacterial membrane disruption. Molecules that intercalate into mitochondrial membranes, such as the mitochondria-targeted antioxidant MitoQ, have been shown to depolarize mitochondria and trigger autophagy^36^. This depolarization stimulates the PINK-1-dependent recruitment of Parkin, which in turn mediates the elimination of mitochondria in lysosomes through mitophagy^43^. In support of this hypothesis, we show that thymol lowers oxygen consumption in live zebrafish and impairs oxygen flux in preparations of freshly isolated mitochondria from mouse hepatocytes. Mechanistically, the induction of mitophagy by thymol depends on PINK-1, while oligomycin—a chemical that inhibits proton flux across the mitochondrial membrane and thereby counteracts membrane depolarization—blunts thymol-induced mitophagy. These results provide robust evidence for a mitochondrial membrane-originating mechanism through which thymol activates autophagy by depolarizing mitochondria, thereby enhancing mitochondrial clearance both in vitro and in vivo.

Mild, transient mitochondrial perturbations activate cellular stress response mechanisms, including autophagy and mitophagy, leading to long-term benefits through a process known as mitohormesis^55,56^. Previous reports show that not all damaged mitochondria can be efficiently cleared by mitophagy unless a certain threshold of damage is crossed and autophagy is activated by independent mechanisms^57,58^. We propose that thymol has a favorable pharmacokinetic profile for achieving mitohormetic effects in vivo. Previous pharmacokinetic studies in animals and humans show that thymol has a rapid *T*_max_ of 0.5 and 1.97 h, respectively, followed by relatively fast clearance, with a half-life of 10.2 h in humans^32,59^. This prompt clearance of circulating thymol suggests that cells are only transiently exposed to its effects. As a result, mitophagy is primarily activated in damaged mitochondria, while autophagy is not activated in healthy mitochondria with normal electrochemical coupling. Rapid absorption in the duodenum further suggests that oral intake leads to transient peaks in thymol and its conjugates, reaching levels sufficient to activate autophagy in tissues before they are cleared^60^. While the exact pharmacokinetic profile and pharmacodynamic mechanism of action of thymol on mitochondrial turnover remain to be established, its common use in traditional medicine supports its safety in humans^61^.

In metabolic dysfunction-associated fatty liver disease, autophagy is known to hydrolyze triacylglycerol from lipid droplets^40^. Mitophagy concomitantly increases the turnover of defective mitochondria, thereby facilitating fatty acid oxidation and efficient metabolic coupling, as well as protecting hepatocytes against aberrant liver fat accumulation^62^. Our findings show that chronic administration of thymol to mice fed a HFD resulted in a reduction in liver lipid deposition and lower levels of P62 protein. Thymol did not affect body weight, which aligns with the potential for caloric restriction mimetic effects independent of weight loss.

Next, we tested the ability of *C. elegans* to adapt to heat stress. Autophagy has been reported to promote thermotolerance due to its role in the removal of protein aggregates that form when cellular temperatures rise to toxic levels^63^. Thymol efficiently preserved activity in response to heat shock, suggesting that autophagy is the underlying protective mechanism. Finally, we showed that thymol-treated worms had increased motility with age, accompanied by a lower degree of myofiber damage, suggesting protection against the age-related decline in body wall muscle function and thereby preserving mobility. Based on these results, we turned to SAMP8 mice, which are characterized by the occurrence of protein aggregates in the brain and skeletal muscle, among other tissues^50^. Thymol demonstrated a remarkable restoration of motor performance, including improvements in strength and endurance. The functional benefits were accompanied by a lower epigenetic age determined using a pan-tissue clock readout, suggesting a potent antiaging effect at the tissue level and its relevance for translation. A limitation of our study is the use of male mice across the different in vivo experiments, a choice made due to the availability constraints of aged animals. Although testing the efficacy of thymol and carvacrol in females is an important next step, the consistent effects observed in zebrafish larvae (a stage before sex determination) and in the hermaphrodite *C. elegans* suggest a largely sex-independent mechanism.

While deploying different species and experimental models is a strength of this work, the approach of using zebrafish larvae has some limitations. Our discovery paradigm is likely to miss compounds that depend on signaling pathways not yet established during early larval stages, as well as those specific to old age. Other limitations include the difficulty in deciding doses for oral efficacy in preclinical models, a problem that we mitigated by relying on the known pharmacokinetics of thymol in humans and rodents.

Terpenoids and polyphenolic compounds are secondary plant metabolites enriched in diets characterized by a high contribution of fruits, vegetables, herbs and spices^64^. Some of these noncaloric phytonutrients are proposed to stimulate autophagy and mitophagy, potentially forming part of the overall health impact of these diets through the cumulative effects of bioactive mixtures^65^. Analyses of dietary habits among people living in areas with a high percentage of centenarians indicate that individuals with extraordinary healthspans consume diets rich in mitohormetic phytochemicals^66^. The limited knowledge of the overall intake doses of thymol from aromatic plants makes it challenging to perform a systematic analysis of its contribution to long-term benefits through diet. Nonetheless, carvacrol- and thymol-containing plants have been used in traditional medicine for their anti-inflammatory, antioxidant and bactericidal properties, although their mechanism of action has remained largely elusive^67,68^.

Secondary plant metabolites have numerous biological functions, including symbiotic advantages such as producing colors and flavors, conferring resistance to stressors (such as drought, heat and radiation) and possessing antipathogenic properties, among other functions that may explain their bioactivity in humans. In fact, a surprising number of plant bioactives have inhibitory or modulatory effects on cellular bioenergetic functions in eukaryotic cells. For example, biguanides from the French lilac (*Galega officinalis*) inhibit mitochondrial respiratory complex I^69,70^ and have served as the lead structures for the antidiabetic medication metformin. Resveratrol and related flavonoid polyphenols from grapes and berries have been shown to bind to and inhibit F_1_-ATPase, slowing ATP synthesis^71^. Here, we demonstrate that thymol activates autophagy and mitophagy through a mechanism initiated at the level of mitochondrial bioenergetics, which may be related to the bactericidal properties of thymol as part of its original function in pathogen defense. Thus, the activation of cellular stress pathways by thymol, and potentially other secondary plant metabolites that target bacterial membranes, may reflect moonlighting functions in mitochondrial bioenergetics, autophagy and mitophagy when ingested and absorbed into mammalian cells.

Beyond the induction of autophagy and mitophagy at the mitochondrial level, pleiotropic effects likely exist. For example, thymol’s effects on bacteria could influence the gut microbiome, which mediates positive health outcomes from fasting and metformin treatment^72,73^. While our cellular experiments demonstrate a cell-autonomous effect on autophagy and mitophagy, the microbial activity of thymol may influence the composition of the host microbiome. Interestingly, oregano essential oil is used in animal feed to lower intestinal inflammation and improve immunity by modulating the composition of intestinal microbiota^74,75^. Transcriptional profiling in MAFs and within a systemic context in skeletal muscle biopsies from SAMP8 mice showed an enrichment of gene signatures compatible with a reduced inflammatory response. Additional studies are warranted to assess the effect of thymol on microbiota, particularly as thymol is believed to be readily absorbed in the upper gastrointestinal tract and may not reach the distal small intestine^76^.

In summary, this study broadens the mechanistic understanding of the health benefits mediated by oregano and thyme terpenoids and shows a potential link between their functions as secondary metabolites in plants and their effects on mitochondrial bioenergetics, autophagy and mitophagy in eukaryotic cells. Thymol is present at high concentrations in common oregano and thyme species and can be enriched in oregano essential oils, making it a relatively simple and cost-effective ingredient for nutrition-based prevention. A range of medical conditions are additional avenues to explore, including neurodegenerative diseases characterized by proteotoxicity, such as Parkinson’s disease and Alzheimer’s disease, as well as the spectrum of inherited mitochondrial diseases.

## Methods

### Antibodies and reagents

A list of antibodies and reagents used in this study is available in the Reporting Summary and Supplementary Table 7.

### Zebrafish husbandry and generation of *Tg(actc1b:ZsGreen-map1lc3;cryaa:TdTomato)* zebrafish line

Adult zebrafish of the AB line were raised at 28 °C under standard conditions. All procedures followed the Swiss and EU ethical guidelines and were approved by the ethics committee of the Canton of Vaud (permits VD3177 and VD3546). Transgenic *Tg(actc1b:ZsGreen-map1lc3;cryaa:TdTomato)* zebrafish were generated using I-SceI meganuclease-mediated insertion into AB embryos at the one-cell stage. The construct included the zebrafish *map1lc3* gene tagged with ZsGreen at the 5′ end under the control of the skeletal muscle-specific promoter *actc1b*. Information on the oligonucleotide sequences based on the zebrafish reference genome GRCz11 is provided in the Supplementary Methods. The line is registered at the Zebrafish Model Organism Database (ZFIN) central repository under the designation *Tg(actc1b:ZsGreen-map1lc3;cryaa:TdTomato*)^nei014^ and can be found at https://zfin.org.

### Mice

All experiments adhered to Swiss regulations on animal experimentation and the European Community Council Directive 2010/63/EU. All procedures were approved by the Nestlé Ethical Committee (ASP-16-38-EXT), the Office Vétérinaire Cantonal Vaudois (VD2770, VD3195 and VD3484), and the Directorate General for Animal Health and Veterinary Drugs of the Italian Ministry of Health (authorization nos. 924/2021-PR and 885/2020-PR). Mice were housed under standard conditions with ad libitum access to food and water. C57BL/6 wild-type mice were purchased from Janvier Labs and were fed either a standard diet (D12450J, Research Diets) or a HFD (D12492, Research Diets). Mito-QC mice were kindly provided by I. Ganley, and SAMP8 AKR/J mice were obtained from Envigo. Thymol was dissolved in corn oil and administered orally. The dose was selected using allometric scaling based on previous pharmacokinetic and pharmacodynamic studies in mice and humans^32,67^. For the acute studies, 10-week-old male mito-QC or wild-type mice on a C57BL/6 background received thymol at a dose of 20 mg per kg body weight, followed by a second dose 14 h later. Two hours after the second dose, the mice were killed. Organs from mito-QC mice were postfixed in 3.7% paraformaldehyde and 200 mM HEPES (pH 7.0) at 4 °C for 24 h. Tissues were then cryopreserved in 30% sucrose/PBS at 4 °C for 2 days, frozen in chilled isopentane on dry ice and cryosectioned at 8 µm. For chronic treatments, 10-week-old C57BL/6 or 8-month-old SAMP8 male mice received thymol at a dose of 20 mg per kg body weight per day or vehicle (corn oil). The mice were killed at the end of the treatments, and tissues were collected for analysis.

### *C. elegans* strains and RNAi experiments

The following *C. elegans* strains were used in this study: wild-type N2, DA2123 (*adIs2122[lgg-1p::GFP::lgg-1+rol-6(su1006)]*), CF1038 (*daf-16(mu86)*), RB2547 (*pink-1(ok3538)*), TG38 (*aak-2(gt33)*), QV225 (*skn-1(zj15)*), RW1596 (*stEx30[p*_*myo-3*_*GFP::MYO-3 + rol-6(su1006)]*) and IR2539 (*unc-119(ed3); Ex[pmyo‐3TOMM‐20::Rosella; unc‐119(+)]*). All strains were obtained from the *Caenorhabditis* Genetics Center (funded by the National Institutes of Health Office of Research Infrastructure Programs P40 OD010440), except for the strain expressing the mtRosella biosensor, which was a kind gift from the Tavernarakis laboratory in Greece. For RNAi experiments, *Escherichia coli* HT115 bacteria were transformed with either the *pink-1* (RNAi) construct or the empty PL4440 vector. Bacteria were grown overnight in liquid cultures containing ampicillin and tetracycline, followed by subculturing in liquid medium with ampicillin. Cultures were mixed with IPTG (2 mM), seeded onto plates and dried overnight. The next day, the animals were placed on the plates. HT115 bacteria expressing *pink-1* (RNAi) were obtained from the Ahringer RNAi library (ID II-4K04).

### Cell lines

Human lymphocytic T Jurkat cells (clone E6.1, ATCC, TIB-152) were used to measure autophagic flux in vitro. Cells were grown in complete RPMI (Thermo Fisher Scientific, 11875093) under standard conditions. For the experiment, cells were washed, counted and incubated at 1 × 10^5^ cells per well in RPMI plus 10% human AB serum (GemCell, GeminiBio, 100-512) in duplicate in a 96-well plate with either rapamycin (Sigma-Aldrich, C2920), thymol (Sigma-Aldrich, 16254), carvacrol (Sigma-Aldrich, W224502), FCCP (Sigma-Aldrich, C2920), or 0.5% DMSO (Sigma-Aldrich, D8418) as the vehicle.

Primary MAFs were obtained from C57BL/6J or mito-QC mice. Biopsy tissues were cut into small pieces with a scalpel blade and distributed on coated dishes with 1.5 ml DMEM high glucose (Thermo Fisher Scientific, 10569010) supplemented with 20% FBS (Thermo Fisher Scientific, 100-18B). Thymol (100 μM), carvacrol (100 µM), valinomycin (1 µM) (Sigma-Aldrich, V0627) and oligomycin (1 nM) (Sigma-Aldrich, O4876) were each dissolved in DMSO, added to the cell medium, and left for 24 h.

### Procedures in zebrafish including small-molecule treatments, high-content imaging and respirometry

Three transgenic embryos per well were placed in 96-well plates and treated with compounds diluted in DMSO. After 16 h, the embryos were anesthetized with 0.016% tricaine (Sigma-Aldrich, E10521) and imaged using the ImageXpress Micro Confocal High-Content Imaging System (Molecular Devices). Tricaine was then washed out, and the embryos were treated with 100 mM NH_4_Cl (Sigma-Aldrich, 09718) for 4 h before being imaged again. For automated imaging, larval images were initially acquired using a 4× objective and analyzed with MetaXpress software (version 6.7.2). A custom Python code has been developed for the automated detection of larvae. The script records the coordinates of an area of the body at a fixed distance from the edges of the tail and the head. Twenty-six Z stacks of each area are acquired using a 20× objective. For the identification and quantification of ZsGreen-LC3 puncta, two-dimensional projections were analyzed. Images were processed as follows: individual Z stacks were projected onto single images using maximal projection. The projection was subsequently smoothed using serial operations, and this smoothed image was then subtracted from the maximal projection to remove the fish background from the puncta signal, resulting in a subtracted image. The smoothed image was then binarized to create the pre-fish body mask, which was cleaned using area filtering. The final object was then shrunk to represent the fish body mask. The mean intensity of the smoothed image covered by the fish body was measured and used to binarize the subtracted image and create the pre-LC3 punctate mask. Finally, an LC3 puncta mask was created by keeping the pre-puncta objects encompassed within the fish body mask. Morphological measurements were recorded from the two final masks. The LC3 punctate total area relative to body surface area was used as a parameter for quantification. Python and MetaXpress codes have been deposited on GitHub and are accessible at https://github.com/giulializzo/zebrafish_high_content_imaging.git.

The OCR in zebrafish larvae was measured using the Seahorse XF24 instrument (Seahorse Bioscience). Larvae were placed in a 24-well islet microplate (XF24 Islet Capture FluxPak no. 101174-100, Seahorse Bioscience), maintained with an islet capture screen and overlaid with 600 µl of egg water without air bubbles. Larvae were allowed to equilibrate for 10 min and maintained at 28.5 °C throughout the entire experiment. Respiration rates were determined every 5 min. Basal respiration was calculated as the average of the last three measurement points (from 40 to 52 min) before the injection of FCCP to a final concentration of 0.8 µM. Uncoupled respiration was determined by the maximum value observed after FCCP injection. Larvae that did not respond to FCCP and exhibited OCR values lower than basal respiration were excluded from the analysis. Rotenone (Sigma-Aldrich, R8875) and antimycin A (Sigma-Aldrich, A8764) were injected at concentrations of 1 µM and 1 µg l^−1^, respectively, to inhibit the electron transport chain, with residual OCR accounting for nonmitochondrial respiration.

### SDS–PAGE and immunoblotting

Total proteins were extracted from mouse tissues or from pools of 20 zebrafish larvae at 3 dpf, as described in the Supplementary Methods. For SDS–PAGE, lysates were diluted, mixed with NuPAGE LDS sample buffer and a sample reducing agent (Thermo Fisher Scientific, NP0008 and NP0009), and heated at 70 °C for 10 min in heating blocks. Proteins (40 µg) were loaded onto precast NuPAGE Bis–Tris 1.0 mm Midi Protein Gels (Thermo Fisher Scientific, WG1402 and WBT01020), and electrophoresis was performed for 90 min at 130 V. Gels were incubated in 20% ethanol for 10 min and transferred onto PVDF membranes using the iBlot2 instrument (Thermo Fisher Scientific). Membranes were rehydrated in TBS–Tween (Thermo Fisher Scientific, 28360) and blocked with 5% skim milk in TBS–Tween for 1 h at room temperature. Primary antibodies were incubated at 4 °C. The following primary antibodies were used: rabbit polyclonal anti-AMPKα (1:1,000, Cell Signaling, 2532), rabbit monoclonal anti-phospho-AMPKα (1:1,000, Cell Signaling, 2535), mouse monoclonal anti-CHOP (1:1,000, Abcam, Ab11419), rabbit polyclonal anti-CLPP (1:1,000, Cell Signaling, 14181), rabbit monoclonal anti-GAPDH (1:5,000, Abcam, Ab181602), mouse monoclonal anti-HSC70 (1:10,000, Santa Cruz Biotechnology, sc-7298), rabbit polyclonal anti-HSP60 (1:1,000, Abcam, Ab46798), rabbit polyclonal anti-LC3 (1:1,000, Novus Biologicals, NB100-2220), rabbit monoclonal anti-Parkin (1:1,000, Abcam, Ab77924), rabbit polyclonal anti-PINK-1 (1:1,000, Novus Biologicals, BC100-494), mouse monoclonal anti-S6 ribosomal protein (1:1,000, Cell Signaling, 2317), rabbit polyclonal anti-phospho-S6 (1:1,000, Cell Signaling, 2211), mouse monoclonal anti-SQSTM1/P62 (1:1,000, Abnova, H00008878-M01), rabbit polyclonal anti-phospho-ubiquitin (S65) (1:1,000, Boston Biochem, A110), rabbit polyclonal anti-VDAC-1 (1:1,000, Abcam, Ab15895), rabbit monoclonal anti-vinculin (1:5,000, Abcam, Ab219649) and total OXPHOS Blue Native WB Antibody Cocktail (1:1,000, Abcam, Ab110412). Anti-rabbit or anti-mouse IgG H&L (HRP) secondary antibodies (1:10,000, Thermo Fisher Scientific, 31460 and 31430) were incubated for 1 h at room temperature and detected using an enhanced chemiluminescence reagent (Thermo Fisher Scientific, 32106).

### In vitro autophagy assay

To measure autophagy in Jurkat cells, we used the Guava LC3 antibody-based assay kit (Luminex, FCCH100171), following the manufacturer’s instructions. The kit includes reagent A (an inhibitor of lysosomal activity), reagent B (a permeabilization solution) and an anti-LC3 antibody conjugated with FITC (clone 4E-12). Half of the replicates were incubated with reagent A for 30 min before the end of the treatment to block lysosomal degradation of LC3 vesicles, thereby measuring autophagic flux. Cells were then transferred to a 96-well V-bottom plate, permeabilized with reagent B to remove the cytoplasmic form of LC3 (LC3-I) and incubated with the FITC-conjugated LC3 antibody. Samples were then analyzed using a Becton Dickinson LSR Fortessa SORP analyzer. Offline analyses were performed using FCS Express Software (De Novo Software), with results expressed as the median fluorescence intensity in the FITC channel.

### Measurement of MMP and mitochondrial superoxide

MMP and mitochondrial superoxide measurements in Jurkat cells were conducted using flow cytometry. For the MMP, JC-10 was used according to the manufacturer’s instructions (Enzo Life Sciences, ENZ-52305). Cells were treated with thymol, carvacrol or FCCP for 30 min, and JC-10 staining was added 15 min before analysis. For mitochondrial superoxide measurements, Jurkat cells were incubated with MitoSOX Red (5 µM) (Thermo Fisher Scientific, M36008) for 20 min before acquisition. Samples were analyzed using a Becton Dickinson LSR Fortessa SORP flow cytometry analyzer. Offline analyses were performed using FCS Express Software (De Novo Software). The gating strategy is described in the Supplementary Methods.

In MAFs, the MMP was assessed by TMRM and MitoTracker costaining. Thymol or carvacrol (100 μM) was added to the medium and left for 24 h. Untreated cells received an equal volume of DMSO. Oligomycin (1 nM) was used to prevent proton efflux and depolarization. For MMP measurement, cells were grown in a 24-well glass-bottom plate in the presence of conditioned medium for 24 h, then costained with TMRM (20 nM) (Thermo Fisher Scientific, T668) and MitoTracker Deep Red (100 nM) (Thermo Fisher Scientific, M22426). Following a 30-min incubation in the dark at 37 °C, the medium was replaced with dye-free conditioned medium. TMRM and MitoTracker signals were analyzed using a TCS SP8 confocal microscope (Leica Biosystems), as described in the Supplementary Methods.

### Mitochondria isolation from mouse liver

After dissection, liver tissue was rapidly cut into small pieces with scissors and placed in ice-cold medium A (0.32 M sucrose, 10 mM Tris–HCl, 1 mM EDTA; pH adjusted to 7.4). The pieces were transferred into a 5-ml Dounce homogenizer (glass–Teflon) at a ratio of 5 ml of medium A per 1 g of tissue. After four to six strokes at 600 rpm on ice, the suspension was transferred into a 15-ml Falcon tube and centrifuged at 1,000*g* for 5 min at 4 °C. The supernatant was transferred into 2-ml microtubes and centrifuged at 12,000*g* for 10 min at 4 °C. The pellet containing mitochondria was resuspended in 1 ml of ice-cold medium A. The mitochondrial protein concentration was then determined using the BCA method (Thermo Fisher Scientific, 23225).

### Respirometry in isolated mitochondria

The OCR in mitochondria isolated from mouse livers was measured using high-resolution respirometry (OROBOROS Oxygraph-2k, Oroboros Instruments). For respirometry analysis, 150 µg of crude mitochondrial extract was added to modified MiR05 buffer (110 mM sucrose, 0.5 mM EGTA, 3 mM MgCl_2_, 20 mM taurine, 10 mM KH_2_PO_4_, 20 mM HEPES and 0.1% BSA essentially fatty acid free) in a 2-ml chamber at 37 °C. Respiration in isolated mitochondria was determined using SUIT (substrate–uncoupler–inhibitor titration) protocols, with modifications. Pyruvate (Sigma-Aldrich, P2256, 5 mM), glutamate (Sigma-Aldrich, G1626, 10 mM) and malate (Sigma-Aldrich, M1000, 2 mM) were used as substrates for inducing complex I respiration in the presence of ADP (Sigma-Aldrich, A5285, 1 mM). A 2-µl volume of thymol solution (50 mM thymol in ethanol) was repeatedly injected to reach incremental concentrations of thymol in the respirometry chamber. The second (control) chamber was injected with the same volume of carrier (ethanol) alone. After a sufficient number of titration steps, respiration was halted by the addition of the complex I inhibitor rotenone (Sigma-Aldrich, R8875, 0.5 µM). Values obtained were normalized to the baseline respiration measured in the control chamber.

### RNA extraction and QuantSeq 3′ mRNA sequencing

Total RNA was extracted as described in the Supplementary Methods. Total RNA (75 ng; 8.9 < RNA quality number < 10) was used to generate the libraries using the QuantSeq 3′ mRNA-Seq Library Prep Kit FWD for Illumina (Lexogen, 015.384) following 20 cycles of PCR amplification. Libraries were quantified using the Quant-iT PicoGreen kit (Invitrogen, Q33140) on a FilterMax F3. The size pattern was assessed using the Fragment Analyzer 96 system with the High Sensitivity NGS Fragment Analysis Kit (Agilent Technologies, DNF-474-0500). Libraries were pooled at an equimolar ratio at a concentration of 40 nM, and sequencing was performed by loading a concentration of 650 pM with a P3 50 kit for 65 cycles on an Illumina NextSeq 2000 platform (Illumina) according to the manufacturer’s instructions.

### Body composition and OGTT

Body composition was measured using nuclear magnetic resonance with the Minispec device (Bruker). Lean body mass and body fat were recorded and normalized to total body weight. OGTT was performed on mice that had been fasted for 6 h. Tail vein glucose levels were measured using a Contour glucometer (Ascensia Diabetes Care) immediately before glucose administration and at 15, 30, 60, 90 and 120 min after glucose administration (1 g glucose per kg body weight). Plasma insulin levels before glucose administration and at 15, 30 and 60 min after glucose administration during the OGTT procedure were quantified from heparinized plasma samples using an ultrasensitive ELISA kit (Crystal Chem, 90080).

### Liver triglyceride measurements

The Triglyceride-Glo Assay kit (Promega, J3160) was used to measure triglyceride levels in mouse liver homogenates. Homogenates were prepared from 40 mg of liver tissue in 1 ml ice-cold PBS, using a TissueLyser II (Qiagen) at a frequency of 30 oscillations per second for 2 min. The homogenates were subsequently centrifuged at 1,000*g* at 4 °C to remove cellular debris. The supernatant was used for the triglyceride assay according to the manufacturer’s instructions.

### Pharmacological treatment of *C. elegans* on plates and chips

For experiments on plates, *C. elegans* strains were cultured at 20 °C on nematode growth medium (NGM) agar plates seeded with the *E. coli* strain OP50. Thymol and carvacrol were mixed into the agar medium before the plates were poured. DMSO was used as a control (maximum concentration of 0.4% for 100 µM). Fresh plates were prepared weekly, and animals were transferred to freshly seeded plates daily after reaching the L4 stage.

For pharmacological treatment on chips, worms were exposed to carvacrol, thymol or 1% DMSO in liquid NGM medium with heat-killed *E. coli* OP50 (20 mg ml^−1^). Compounds were dissolved in DMSO and prepared at a 2× concentration in liquid NGM. On the day of treatment, the 2× stock was diluted to the final concentration with liquid NGM containing heat-killed *E. coli* OP50. Once the animals reached the L4 stage, they were loaded into microfluidic chips (Infinity Chips, NemaLife) and maintained on a diet of heat-killed *E. coli* OP50 for 24 h. On day 1 of adulthood, worms were treated with the appropriate compound or 1% DMSO. Medium exchange was performed daily to maintain consistent exposure until the end of the assays.

### Quantification of autophagy and mitophagy in *C. elegans*

The number of autophagosomes was assessed in the DA2123 *C. elegans* strain that expresses GFP::LGG-1, with autophagosomes detected as GFP^+^ puncta. Animals were collected, washed to remove bacteria and anesthetized using metamizole at a final concentration of 20 mM. The animals were then placed on slides and observed under the 40× lens of a Leica DM5500 upright microscope. Mitophagy was measured using a strain that expresses the mtRosella biosensor in the mitochondria of body wall muscle cells. Mitophagy was calculated as the ratio of pH-insensitive DsRed to pH-sensitive GFP, as previously described^77^.

### Healthspan and thermotolerance assays in *C. elegans* on chips

For the healthspan assay, one 90-s video per chip was recorded for each time point. The activity score analysis involved determining the fraction of pixels (representing a worm within a bounding box) that changed over 30 s. Highly active animals that completely moved their bodies out of the bounding box were assigned a score of 1, whereas completely inactive animals received a score of 0. Animals with intermediate scores still had a portion of their bodies remaining in the bounding box after 30 s. For thermotolerance assays, L4 animals and *C. elegans* carrying deletions of *daf-16*, *pink-1*, *aak-2* and *skin-1* were loaded into microfluidic chips at 20 °C with NGM. On day 1 of adulthood, the medium was exchanged with NGM containing the appropriate treatment, and the animals were allowed to grow until day 3 of adulthood. Subsequently, the animals were exposed to a 37 °C heat shock for 4 h, followed by recovery and scoring for the number of alive animals in the chips.

### Motility and lifespan assays in *C. elegans* on plates

The mean speed and distance crawled by *C. elegans* on the plate were measured on day 10 of adulthood using the Movement Tracker software, as previously described^78^. Lifespan tests were performed at 20 °C, and the worms were monitored daily for survival. Worms were considered to have died if they failed to respond to gentle prodding with a platinum wire. Lifespan data were recorded until all the worms had died. Lifespan metrics were calculated and analyzed using the Kaplan–Meier method.

### Analysis of myofibril damage in *C. elegans*

RW1596 animals were grown on NGM plates seeded with *E. coli* OP50 bacteria and containing either thymol, carvacrol or vehicle. The animals were maintained on these plates until day 10 of adulthood, after which they were collected, washed to remove bacteria and anesthetized with tetramisole at a final concentration of 20 mM. They were then placed on slides and observed under the 40× lens of a Leica DM5500 upright microscope.

At least 200 muscle cells were counted for each condition. Muscle cells containing broken myofibrils or myofibrils with abnormal orientation were classified as damaged, while those with intact myofibrils showing normal, parallel orientation were classified as normal. Finally, the percentage of damaged myofibers relative to the total number of myofibers was calculated.

### Measurement of biological age

DNA extraction was performed on 100 mg of muscle tissue using the Qiagen DNeasy Blood and Tissue kit, following the manufacturer’s recommendations. DNA was quantified using DropSense technology (Unchained Labs). Bisulfite conversion was performed on 500 ng of DNA using the Zymo EZ DNA methylation kit, following the manufacturer’s instructions. Converted DNA (200 ng) was used to proceed with the Infinium HD Methylation Assay Protocol, following the manufacturer’s recommendations. DNA methylation data were generated using custom arrays (HorvathMammalMethyl40, Illumina) and scanned on an iScan reader (Illumina). Biological age was quantified based on a pan-tissue epigenetic clock algorithm^52^.

### Behavioral analysis in mice

Endurance was evaluated using a treadmill apparatus (Ugo Basile) with a gradually accelerating protocol, starting at 5 rpm for 2 min and increasing to 50 m s^−1^ over 2,700 s. The test was terminated by exhaustion, defined as ten falls per minute in the motivational air puff.

Weakness was assessed by measuring forelimb grip strength using a grip strength meter (Ugo Basile). Mice grasped the grid with their forelimbs and were gently pulled away by holding the base of their tails. Measurements were recorded in triplicate, and the average was used for the analysis.

The Y maze task was used to measure spontaneous alternation behavior. Mice were introduced to the center of the maze and allowed to explore the three arms for 6 min. The number of triads was recorded to calculate the percentage of alternation. An entry was defined when all four limbs were within an arm.

A rotarod apparatus (Ugo Basile) was used to assess coordination skills. After two acclimation sessions, the apparatus was set to a starting velocity of 4 rpm and an acceleration rate of 20 rpm per minute. Mice underwent three trial sessions at least 20 min apart, and the latency to fall was recorded for each mouse.

### Immunohistochemistry

For histochemical analysis, tissues were frozen in liquid nitrogen-precooled isopentane. The tibialis anterior muscle was cut into 12-μm sections at −25 °C, and the sections were thaw-mounted on slides. The slides were dried at room temperature and stained using a monoclonal anti-laminin antibody (Sigma-Aldrich, L9393) diluted 1:100 and a goat anti-rabbit IgG H&L (Alexa Fluor 405) secondary antibody (Thermo Fisher Scientific, A48254) diluted 1:500 for detection. Livers were cryodissected at 8 µm and fixed overnight in 4% paraformaldehyde. The ORO stock stain was prepared by dissolving 0.5 g of ORO (Sigma-Aldrich, O0625) in 100 ml of isopropanol. The working solution was prepared by diluting 30 ml of the stock with 20 ml of distilled water. Liver tissues were cut into 2-mm-thick slices, fixed in formalin, paraffinized and hydrated. After deparaffinization and rehydration with xylene and ethanol, the slides were stained with ORO working solution for 15 min, rinsed with 60% isopropanol and lightly stained with hematoxylin for 4 min. After rinsing with distilled water, the slides were mounted. Lipid droplets were stained red and assessed using an Olympus VS120 slide scanner. The size of lipid droplets per field was calculated based on the average size of lipid droplets in ten fields from each mouse liver slide, using the multipoint function of ImageJ.

### Analysis of differentially expressed genes

Raw counts were mapped to the mouse reference genome (GRCm38-101) using STAR (v.2.5.3) and counted using HTSeq-count (v.0.6.1). Data were checked for quality, and no outliers were detected in the principal component analysis. A filter was applied to select only genes with a minimum of 30 reads in at least six samples (corresponding to a threshold of 3.809 on the count per million values), and nonannotated genes were discarded. A total of 10,697 features were retained using these filtering criteria. To account for composition bias between libraries, we applied TMM (trimmed mean of M-value) normalization^79^. Differential expression analysis between groups was performed using edgeR (v.4.2.1), which estimates gene-wise negative binomial dispersions by calculating the adjusted profile log-likelihood for each gene and maximizing it using the weighted likelihood empirical Bayes method^80^. A quasi-likelihood negative binomial generalized log-linear model was fitted to the count data, and a gene-wise empirical Bayes quasi-likelihood *F* test was conducted for each comparison. Resulting *P* values were adjusted for multiple testing using the Benjamini–Hochberg correction method.

### Gene set enrichment analysis

Gene set enrichment analysis was performed using the Camera function in the edgeR package^81^, which conducts a competitive test to assess whether the genes in the set are highly ranked in terms of differential expression relative to genes not in the set, while accounting for intergene correlation. Camera was applied to the hallmark pathways from the mouse MSigDB^82^. The Fry algorithm from edgeR was applied to assess the enrichment of a preselected list of pathways. Fry is a fast approximation of a rotation gene set test that performs self-contained gene set tests to assess whether any of the genes in the set are differentially expressed^83^. *P* values are obtained through simulation, followed by adjustment for multiple testing.

### Mitophagy in mito-QC fibroblasts and mice

For each coverslip containing mito-QC fibroblasts or tissues, ten random images were acquired using a Leica TCS SP8 confocal microscope with a 63× oil immersion objective. Images were processed with ImageJ using a custom macro, as described in the Supplementary Methods.

### Statistics and reproducibility

GraphPad Prism software version 9.0 (GraphPad Software) was used to perform all statistical analyses as indicated in the main text and figure legends, except for the gene expression and pathway analyses. Bar graphs show means ± s.e.m. Box plot elements show the following data: median (center line), upper and lower quartiles (box limits), minimum and maximum values (whiskers), and points. Differences between two groups were assessed using Student’s *t* test. Differences among more than two groups were assessed using one-way analysis of variance (ANOVA). The interaction between two factors was tested using a two-way ANOVA. Data distribution was assumed to be normal, but this was not formally tested. Survival analysis in zebrafish and worms was performed using the Kaplan–Meier method, and pairwise comparisons were conducted using the log-rank test. For high-content imaging, zebrafish larvae that were displaced during acquisition, resulting in undetectable LC3-positive puncta, were excluded from the analysis. No randomization method was used in zebrafish, cell or worm experiments. Mice were randomized using simple randomization based on body weight within the experimental groups. All phenotyping experiments in mice were conducted with the experimenters blinded to the treatment, and data analyses were performed without knowledge of the experimental conditions. Chronic mouse interventions were performed once, but they followed blinding and randomization to ensure reproducibility. For mouse studies, animals that showed weight loss greater than 20%, those found dead in their cages, and those displaying signs of infection and inflammation in their wounds were killed and excluded from the study.

For the worm and zebrafish experiments, the researchers performing the assays were blinded to the treatment. Histopathology, confocal imaging and image analysis were performed in a blinded manner. For all other experiments, blinding was not formally conducted. For short-term thymol supplementation studies in mice, the sample size was determined based on previous publications to detect significant changes in mitophagy or autophagy induction, as measured by the mitophagy index or the LC3-II/LC3-I ratio, respectively^38,84^. For the metabolic dysfunction-associated fatty liver disease model, the sample size was similar to that reported in other studies^85^. For the SAMP8 study, treadmill performance exhibited the greatest variability among all the parameters considered. Based on pilot studies, a 20% decline in motor performance was expected between 8 and 11 months of age. With a study power (*β*) set at 0.80, an *α* error of 0.05, and assumptions of a halt in motor decline from the start of treatment and a 25% mortality rate, a sample size of 15 animals per group was deemed necessary. SAMP8 mice that died before the end of the experiment were excluded from subsequent analyses. For *C. elegans* studies, sample sizes were determined based on previous publications^42,86^. For all representative images showing cells, zebrafish or *C. elegans*, experiments were repeated a minimum of three times independently and checked to ensure the reproducibility of the results. The graphics used in the schemata throughout the paper were created using BioRender.com.

### Reporting summary

Further information on research design is available in the Nature Portfolio Reporting Summary linked to this article.

## Supplementary information

Supplementary Information Supplementary Fig. 1 and Methods.

Reporting Summary

Supplementary Tables Supplementary Tables 1–7.

## Source data

Source Data Extended Data Fig. 1 Uncropped western blots.

Source Data Extended Data Fig. 2 Uncropped western blots.

Source Data Extended Data Fig. 7 Uncropped western blots.

Source Data Fig. 5 Uncropped western blots.

Source Data Fig. 7 Uncropped western blots.

Source Data Fig. 1 Source data.

Source Data Fig. 2 Source data.

Source Data Fig. 3 Source data.

Source Data Fig. 4 Source data.

Source Data Fig. 5 Source data.

Source Data Fig. 6 Source data.

Source Data Fig. 7 Source data.

## Data Availability

All processed data associated with this study are included in the article, extended data figures, supplementary tables and source data files. Unprocessed transcriptomic data based on 3′ QuantSeq analysis have been deposited in the Gene Expression Omnibus (GEO, https://www.ncbi.nlm.nih.gov/geo/) database and are accessible through the identifiers GSE298195 and GSE298196. Epigenetic data were generated through a custom array for mammalian species (HorvathMammalMethyl40, Illumina) and can be obtained from the nonprofit research organization Epigenetic Clock Development Foundation (‘Clock Foundation’, https://clockfoundation.org/) upon reasonable request. For all other datasets underlying the results, no data restriction applies to the raw data generated in this study, and they can be requested from the corresponding authors. Source data are provided with this paper.

## Extended data

**Extended Data Table 1:** Main components of *Origanum Vulgare* essential oil

Compound	%
α-PINENE	1,10
α-THUYENE	1,69
β-MYRCENE	1,52
α-TERPINENE	1,62
γ-TERPINENE	4,71
p-CYMENE	16,39
1-OCTEN-3-OL	1,50
TERPINENE-4-OL	1,33
β-CARYOPHYLLENE	2,40
THYMOL	5,14
CARVACROL	55,82

## Code availability

For the quantification of confocal images of zebrafish larvae, an automatic workflow has been generated using MetaXpress software version 6.7.2, as illustrated in Extended Data Fig. 1 and described in the Methods. Custom Python and MetaXpress codes have been deposited on GitHub and are accessible via the following link: https://github.com/giulializzo/zebrafish_high_content_imaging.git. No new code was generated for the quantification of gene expression and DNA methylation analyses.
