# Supplementary material for: Herbal terpenoids activate autophagy and mitophagy through modulation of bioenergetics and protect from metabolic stress, sarcopenia and epigenetic aging
Source: Nat Aging. 2025 Sep 24;5(10):2003–21. doi: 10.1038/s43587-025-00957-4 (PMC12532568; doi:10.1038/s43587-025-00957-4)
Supplement: Supplementary file 1 — Supplementary Fig. 1 and Methods. [file 43587_2025_957_MOESM1_ESM.pdf]

nature aging

Supplementary information https://doi.org/10.1038/s43587-025-00957-4

Herbal terpenoids activate autophagy and mitophagy through modulation of bioenergetics and protect from metabolic stress, sarcopenia and epigenetic aging

In the format provided by the authors and unedited

SUPPLEMENTARY FIGURES

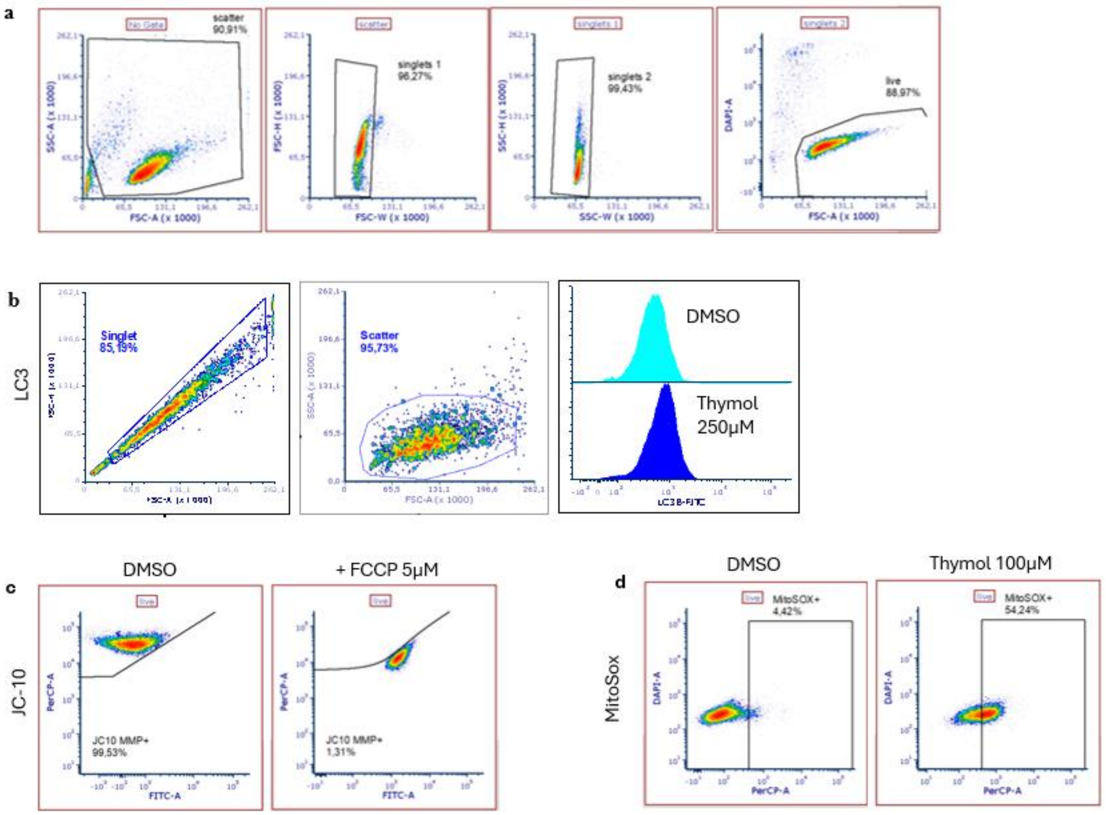

Supplementary Fig. 1 Representative gating strategy for flow cytometric analyses

a Gating on size and granularity, single cells and live cells using DAPI. **b** Gating for autophagy flux measurement. A first gate was drawn including single cell populations and to exclude out-of-scale events (Singlet). Then a Scatter Gate was drawn to select cell population in a forward/side scatter (FSC/SSC) plot. MFI values for the LC3 marker were obtained by histogram analysis in the gate "Scatter" (right panel, histograms for control (DMSO) and treated cells with thymol 250µM. **c** Gating on JC-10 green positive cells treated with DMOS or FCCP.

SUPPLEMENTARY METHODS

Generation of *Tg(actc1b:ZsGreen-map1lc3;cryaa:TdTomato)* zebrafish line

Transgenic zebrafish were generated using I-SCEI meganuclease-mediated insertion into AB embryos at the one-cell stage. *ZsGreen-map1lc3* oligonucleotide was synthesized by GeneArt (Thermo Fisher Scientific) and cloned into a plasmid for stable expression under the *actc1b* promoter. For rapid selection of transgenic animals, the plasmid carried an eye-marker cassette harbouring *TdTomato* under the control of the *cryaa* (alpha-crystallin A chain) promoter in reverse direction. The sequence of *ZsGreen-map1lc3* oligonucleotide was the following:

ATGGCCCAGTCCAAGCACGGCCTGACCAAGGAGATGACCATGAAGTACCGCATGGAGGGCTGCGTGGACGGCCA
CAAGTTCGTGATCACCGGCGAGGGCATCGGCTACCCCTTCAAGGGCAAGCAGGCCATCAACCTGTGCGTGGTGGA
GGGCGGCCCCTTGCCCTTCGCCGAGGACATCTTGTCCGCCGCCTTCATGTACGGCAACCGCGTGTTCACCGAGTAC
CCCCAGGACATCGTCGACTACTTCAAGAACTCCTGCCCCGCCGGCTACACCTGGGACCGCTCCTTCCTGTTCGAGG
ACGGCGCCGTGTGCATCTGCAACGCCGACATCACCGTGAGCGTGGAGGAGAACTGCATGTACCACGAGTCCAAGT
TCTACGGCGTGAACTTCCCCGCCGACGGCCCCGTGATGAAGAAGATGACCGACAACTGGGAGCCCTCCTGCGAGA
AGATCATCCCCGTGCCCAAGCAGGGCATCTTGAAGGGCGACGTGAGCATGTACCTGCTGCTGAAGGACGGTGGC
CGCTTGCGCTGCCAGTTCGACACCGTGTACAAGGCCAAGTCCGTGCCCCGCAAGATGCCCGACTGGCACTTCATCC
AGCACAAGCTGACCCGCGAGGACCGCAGCGACGCCAAGAACCAGAAGTGGCACCTGACCGAGCACGCCATCGCC
TCCGGCTCCGCCTTGCCCGGGGGTGGCTCTGGTGGAGGTAGCATGCCTTCGGAAAAGACATTTAAACAAAGGAG
GACATTTGAGCAGCGGGTGGAGGATGTACGGCTGATCCGGGAACAGCATCCAAACAAGATCCCGGTCATCATCG
AGAGATACAAGGGAGAGAAGCAACTGCCGATTCTTGACAAAACTAAGTTTCTAGTTCCTGACCACGTTAACATGA
GCGAACTTATTAAGATTATCAGGCGACGCCTCCAACTCAACTCCAACCAGGCTTTCTTCCTGCTGGTCAACGGTCAC
AGCATGGTATCTGTGTCCACTGCCATTTCTGAGGTCTATGAACGGGAAAGAGACGAAGACGGCTTTCTGTACATG
GTCTACGCTTCCCAGGAGACATTTGGATTTCAG

Total proteins extraction

Proteins were extracted from zebrafish larvae or mouse tissues using RIPA Buffer (Sigma-Aldrich, R0278) with 1% (V/V) Halt Phosphatase Inhibitor (Thermo Fisher Scientific, 78426) and 1% (V/V) Halt Protease Inhibitor (Thermo Fisher Scientific, 78438). Mouse tissues were homogenized using the gentleMACS™ Octo Dissociator (Miltenyi Biotec) and zebrafish larvae were homogenized with a pestle motor mixer (Argos Technologies). Samples were centrifuged at 13000 x g for 10 min at 4°C and supernatants were used for further analyses. Protein quantification was performed using the BCA assay kit (Thermo Fisher Scientific, 23225).

Total RNA extraction

Total RNA was extracted from snap-frozen MAFs and hindlimb muscles tissues using the Agencourt RNAdvance Tissue Kit (Beckman Coulter, A32646) following the manufacturer's instruction. Total RNA was quantified using the Quant-iT RiboGreen RNA Assay Kit (Invitrogen, R11490) on a FilterMax F3 (Molecular Devices, Sunnyvale) and quality was assessed with Fragment Analyzer-96 using DNF-471-0500 Standard Sensitivity RNA Analysis Kit (Agilent Technologies, DNF-471).

Quantification of mitochondrial membrane potential in Mouse Adult Fibroblasts (MAFs)

Mitochondrial membrane potential was evaluated in MAFs by normalizing TMRM signal to mitochondrial area using ImageJ. A mask around every single cell was manually drawn to analyze the mitochondrial content using Mitotracker signal: Image> Adjust> Threshold. Edit> Selection> Create selection. Analyze> Tools> ROI Manager >Add. The same Threshold was applied to all

images. Mitochondrial mask area was measured: Analyze >Measure> Area. The signal of the TMRM in the mitochondrial mask was measured using: Analyze> Measure> Integrated density. The sum of TMRM integrated density for each stack plane was normalized to the sum of the mitochondrial area.

Quantification of mitophagy

To analyze the mCherry mitolysosomes puncta number and area, both GFP and mCherry images were pre-processed as follow: Process > Subtract Background (Rolling ball radius = 50 pixels), Plugins > Sigma Filter Plus (Radius =2.0 pixels, Use pixels within = 2.0 sigmas, Minimum pixel fraction = 0.2, outlier aware), Process > Enhance Local Contrast (Blocksize = 63, Histogram = 256, Maximum = 2), Process > Math > Gamma (Value = 0.8), Process > Noise > Despeckle. Only for MAFs analyses, an arbitrary value of 25 was add to GFP image: Process > Math > Add (Value = 25). mCherry-positive, GFP-negative puncta were identified subtracting GFP processed image to the mCherrry one: Process > Image calculator (Image 1 = mCherry, Operation = Subtract, Image 2 =GFP), Process > Noise > Despeckle, Image > Adjust > Threshold, Process > Binary > Watershed, Analyze > Analyze Particles. To evaluate mitochondria area, the GFP image was processed as follow: Process > Subtract Background (Rolling ball radius = 50 pixels), Plugins > Sigma Filter Plus (Radius =2.0 pixels, Use pixels within = 2.0 sigmas, Minimum pixel fraction = 0.2, outlier aware), Process > Enhance Local Contrast (Blocksize = 63, Histogram = 256, Maximum = 2), Process > Math > Gamma (Value = 0.8), Process > Noise > Despeckle, Image > Adjust > Threshold, Analyze > Measure.
