# Supplementary material for: Herbal terpenoids activate autophagy and mitophagy through modulation of bioenergetics and protect from metabolic stress, sarcopenia and epigenetic aging
Source: Nat Aging. 2025 Sep 24;5(10):2003–21. doi: 10.1038/s43587-025-00957-4 (PMC12532568; doi:10.1038/s43587-025-00957-4)
Supplement: Supplementary file 2 — Reporting Summary [file 43587_2025_957_MOESM2_ESM.pdf]

nature portfolio

Corresponding author(s): Gabriele Civiletto, Jerome Feige, Philipp Gut

Last updated by author(s): 2025/0729

Reporting Summary

Nature Portfolio wishes to improve the reproducibility of the work that we publish. This form provides structure for consistency and transparency in reporting. For further information on Nature Portfolio policies, see our Editorial Policies and the Editorial Policy Checklist.

Statistics

For all statistical analyses, confirm that the following items are present in the figure legend, table legend, main text, or Methods section.

n/a	Confirmed	
☐	☒	The exact sample size (n) for each experimental group/condition, given as a discrete number and unit of measurement
☐	☒	A statement on whether measurements were taken from distinct samples or whether the same sample was measured repeatedly
☐	☒	The statistical test(s) used AND whether they are one- or two-sided<br>*Only common tests should be described solely by name; describe more complex techniques in the Methods section.*
☐	☒	A description of all covariates tested
☐	☒	A description of any assumptions or corrections, such as tests of normality and adjustment for multiple comparisons
☐	☒	A full description of the statistical parameters including central tendency (e.g. means) or other basic estimates (e.g. regression coefficient) AND variation (e.g. standard deviation) or associated estimates of uncertainty (e.g. confidence intervals)
☐	☒	For null hypothesis testing, the test statistic (e.g. F, t, r) with confidence intervals, effect sizes, degrees of freedom and P value noted<br>*Give P values as exact values whenever suitable.*
☒	☐	For Bayesian analysis, information on the choice of priors and Markov chain Monte Carlo settings
☒	☐	For hierarchical and complex designs, identification of the appropriate level for tests and full reporting of outcomes
☐	☒	Estimates of effect sizes (e.g. Cohen's d, Pearson's r), indicating how they were calculated

Our web collection on statistics for biologists contains articles on many of the points above.

Software and code

Policy information about availability of computer code

Data collection	No new codes were written for this study with the exception of a script for the quantification of autophagosomes in live zebrafish. For the quantification of confocal images of zebrafish larvae, an automatic workflow has been generated using MetaXpress software Version: 6.7.2 as illustrated in Extended Data Fig. 1 and METHODS. Custom Python and MetaXpress codes have been deposited in GitHub and are accessible via the link: https://github.com/giulializzo/zebrafish_high_content_imaging.git.
Data analysis	For the analysis of differentially expressed genes, raw counts were mapped to mouse reference genome (GRCm38-101) using STAR (v.2.5.3) and counted using htseq-count (0.6.1). Differential expression analysis between groups was performed using edgeR (v.4.2.1). Gene set enrichment analysis was performed using CAMERA from the edgeR package querying gene sets annotated in MSigDB version .<br>For all other experimental data analyses were performed as indicated in the main text and figure legends using GraphPad Prism software version 9.0 (GraphPad Software, San Diego, CA, USA).<br>For zebrafish imaging, larvae images were acquired with ImageXpress Micro Confocal High-Content Imaging System (Molecular Devices) and analysed with MetaXpress software, version 6.7.2.<br>Oxygen consumption rate (OCR) in zebrafish larvae was measured using a Seahorse XF24 instrument and software (Seahorse Bioscience).<br>Flow cytometry experiments were carried out with Becton Dickinson LSORP Fortessa analyzer and analyzed with FCS Express Software (De Novo Software)<br>Mitochondrial membrane potential and mitophagy in Mouse Adult Fibroblasts were analyzed using TCS-SP8 confocal microscope (Leica Biosystems) and quantified with ImageJ software<br>Oxygen consumption rate has been measured in liver isolated mitochondria using Oroboros high-resolution respirometry and software (O2k; Oroboros Instruments, Innsbruck, Austria).<br>Liver Oil red O images were acquired using an Olympus VS120 slide scanner and processed with ImageJ software.<br>Mitophagy and autophagy in C.elegans have been quantified by imaging using Leica DM5500 upright microscope and software.

C. elegans movement analyses were performed using the Movement Tracker software (Mouchiroud, L., et al. 2016. DOI: 10.1002/cpns.17), version 1.

For manuscripts utilizing custom algorithms or software that are central to the research but not yet described in published literature, software must be made available to editors and reviewers. We strongly encourage code deposition in a community repository (e.g. GitHub). See the Nature Portfolio guidelines for submitting code & software for further information.

Data

Policy information about availability of data

All manuscripts must include a data availability statement. This statement should provide the following information, where applicable:

- Accession codes, unique identifiers, or web links for publicly available datasets
- A description of any restrictions on data availability
- For clinical datasets or third party data, please ensure that the statement adheres to our policy

All processed data associated with this study are present in the paper, the Extended Data Figures, Supplementary Tables, and Source Data Files. Unprocessed transcriptomic data based on 3' QuantSeq analysis have been deposited at the Gene Expression Omnibus (GEO, https://www.ncbi.nlm.nih.gov/geo/) database and are accessible through the identifiers GSE298195 and GSE298196. Epigenetic data were generated through a custom array for mammalian species (HorvathMammalMethyl40; Illumina) and can be requested upon reasonable request from the non-profit research organization Clock Foundation (https://clockfoundation.org/). For all other data sets underlying the results, no data restriction applies to the raw data generated in this study and can be requested from the corresponding authors.

Research involving human participants, their data, or biological material

Policy information about studies with human participants or human data. See also policy information about sex, gender (identity/presentation), and sexual orientation and race, ethnicity and racism.

Reporting on sex and gender	Not applicable
Reporting on race, ethnicity, or other socially relevant groupings	Not applicable
Population characteristics	Not applicable
Recruitment	Not applicable
Ethics oversight	Not applicable

Note that full information on the approval of the study protocol must also be provided in the manuscript.

Field-specific reporting

Please select the one below that is the best fit for your research. If you are not sure, read the appropriate sections before making your selection.

☒ Life sciences ☐ Behavioural & social sciences ☐ Ecological, evolutionary & environmental sciences

For a reference copy of the document with all sections, see nature.com/documents/nr-reporting-summary-flat.pdf

Life sciences study design

All studies must disclose on these points even when the disclosure is negative.

Sample size	For zebrafish, cell and worm studies sample size was estimated based on (i) the variability observed in pilot studies or in previous related experiments, (ii) the expected effect size, and (iii) the probability to detect a minimal significance level of 0.05. For short-term thymol supplementation studies in mice, sample size was determined based on previous publications to detect significant changes in mitophagy or autophagy as measured by mitophagy index or the LC3-II/LC3-I ratio, respectively (McWilliams, T.G., et al. 2016. DOI: 10.1083/jcb.201603039; Chrisam, M., et al. 2015. DOI: 10.1080/15548627.2015.1098792). For MAFLD model sample size was similar to other studies reported in the literature (Recena Aydos, L., et al. 2019. DOI: 10.3390/nu11092115). For SAMP8 study, treadmill performance exhibited the greatest variability among all parameters considered. Based on pilot studies, a 20% decline in motor performance was expected between 8 and 11 months of age. With a study power (β) set at 0.80, an alpha error (α) at 0.05, and assuming a halt in motor decline from the start of treatment, as well as a 25% mortality rate, 15 animals per group were deemed necessary.
Data exclusions	For rodent studies, mice that showed weight loss higher than 20%, were found dead in cages or displayed signs of infection and inflammation to wounds were sacrificed and excluded from the study.
Replication	Results were confirmed by multiple independent experiments and stringent statistical testing was performed to ensure reproducibility. For all representative images displayed showing cells, zebrafish or C. elegans, experiments were repeated a minimum of three times independently and checked to ensure the reproducibility of results. Chronic mouse interventions were performed once but followed blinding and

randomization to ensure reproducibility.

Randomization

No randomization method was used in zebrafish, cell or worm experiments. Mice were randomized using simple randomization by body weight within experimental groups.

Blinding

For mouse studies, all phenotyping experiments were performed with the experimenters blinded to treatment and data analyses were performed blinded to the conditions of the experiments. For worm and zebrafish experiments, researchers performing the assays were blinded to the treatment. For all the other experiments, blinding was not formally performed.

Reporting for specific materials, systems and methods

We require information from authors about some types of materials, experimental systems and methods used in many studies. Here, indicate whether each material, system or method listed is relevant to your study. If you are not sure if a list item applies to your research, read the appropriate section before selecting a response.

Materials & experimental systems

n/a	Involved in the study	
☐	☒	Antibodies
☐	☒	Eukaryotic cell lines
☒	☐	Palaeontology and archaeology
☐	☒	Animals and other organisms
☒	☐	Clinical data
☒	☐	Dual use research of concern
☒	☐	Plants

Methods

n/a	Involved in the study	
☒	☐	ChIP-seq
☐	☒	Flow cytometry
☒	☐	MRI-based neuroimaging

Antibodies

Antibodies used

The following primary antibodies were used: rabbit polyclonal anti-AMPKα (1:1000, Cell Signaling, 2532), rabbit monoclonal anti-phospho-AMPKα (1:1000, Cell Signaling, 2535), mouse monoclonal anti-CHOP (1:1000, Abcam, Ab11419), rabbit polyclonal anti-CLPP (1:1000, Cell Signaling, 14181), rabbit monoclonal anti-GAPDH (1:5000, Abcam, Ab181602), mouse monoclonal anti-HSC70 (1:10000, Santa Cruz Biotechnology, sc-7298), rabbit polyclonal anti-HSP60 (1:1000, Abcam, Ab46798), rabbit monoclonal anti-Laminin (1:100, Sigma-Aldrich, L9393), rabbit polyclonal anti-LC3 (1:1000, Novus Biologicals, NB100-2220), rabbit monoclonal anti-Parkin (1:1000, Abcam, Ab77924), rabbit polyclonal anti-PINK1 (1:1000, Novus Biologicals, BC100-494), mouse monoclonal anti-S6 ribosomal protein (1:1000, Cell Signaling, 2317), rabbit polyclonal anti-phospho-S6 (1:1000, Cell Signaling, 2211), mouse monoclonal anti-SQSTM1/p62 (1:1000, Abnova, H00008878-M01), rabbit polyclonal anti-phospho-ubiquitin (S65) (1:1000, Boston Biochem, A110), rabbit polyclonal anti-VDAC-1 (1:1000, Abcam, Ab15895), rabbit monoclonal anti-vinculin (1:5000, Abcam, Ab219649), and total OXPHOS Blue Native WB Antibody Cocktail (1:1000, Abcam, Ab110412).

Validation

Antibodies were validated either by the manufacturers or by other research groups:
1. Rabbit polyclonal anti-AMPKα (Cell Signaling, 2535) has been validated by the manufacturer for western blotting in extracts from different cell lines and in other published articles (PMID: 29317208; PMID: 28553939)
2. Rabbit monoclonal anti-phospho-AMPKα (Cell Signaling, 2535): Validated by the manufacturer for western blotting in extracts from different cell lines and cited in other published articles (PMID: 29317208; PMID: 28553939).
3. Mouse monoclonal anti-CHOP (Abcam, Ab11419): Validated by the manufacturer for western blotting in CHOP / DDIT3 knockout cell line and cited in over 235 publications (PMID: 37743418; PMID: 37840563).
4. Rabbit polyclonal anti-CLPP (Cell Signaling, 14181): Validated by the manufacturer for Western blotting in different cell lines including 293T cells, mock transfected (-) or transfected with a construct expressing Myc/DDK-tagged full-length human CLPP protein (hCLPP-Myc/DDK; +).
5. Rabbit monoclonal anti-GAPDH (Abcam, Ab181602): Validated by the manufacturer for western blotting in several cell and tissue lysates and cited in over 2400 publications (PMID: 38050142; PMID: 38125349).
6. Mouse monoclonal anti-HSC70 (Santa Cruz Biotechnology, sc-7298): Validated by the manufacturer for western blotting in several cell and tissue lysates and cited in over 800 publications (PMID: 36791253; PMID: 37082730).
7. Rabbit polyclonal anti-HSP60 (Abcam, Ab46798): Validated by the manufacturer for western blotting in several cell and tissue lysates and cited in over 130 publications (PMID: 38055816; PMID: 37507476).
8. Rabbit polyclonal anti-LC3 (Novus Biologicals, NB100-2220): Validated by the manufacturer for western blotting by genetic and pharmacological strategies and cited in over 1800 publications (PMID: 34315875; PMID: 34622072).
9. Rabbit monoclonal anti-Parkin (Abcam, Ab77924): Validated by the manufacturer for western blotting in PRKN knockout cell line and cited in over 150 publications (PMID: 37416768; PMID: 36880403).
10. Rabbit polyclonal anti-PINK1 (Novus Biologicals, BC100-494): Validated by the manufacturer for western blotting in lysates from different species and from subcellular fractionation, pathogenic mutants, cells transfected with PINK1 siRNA, PINK1 -/- platelets and CCCP treated cells. It has been cited in over 200 publications (PMID: 31908016; PMID: 25083992)
11. Mouse monoclonal anti-S6 ribosomal protein (Cell Signaling, 2317): Validated by the manufacturer for western blotting in lysates from different cell lines. Cited for western blotting in over 100 publications including in zebrafish and mouse species (PMID: 3882049; PMID: 10620464)
12. Rabbit polyclonal anti-phospho-S6 (Cell Signaling, 2211): Validated by the manufacturer for western blotting in extracts from 293 cells, untreated or treated with 20% FBS. Cited for western blotting in over 100 publications (PMID: 31908016; PMID: 25083992)
13. Mouse monoclonal anti-SQSTM1/p62 (Abnova, H00008878-M01): Validated by the manufacturer for western blotting detection against Immunogen. Cited in several publications (PMID: 31908016; PMID: 25083992)
14. Rabbit polyclonal anti-phospho-ubiquitin (S65) (Boston Biochem, A110): Validated by the manufacturer for western blotting by

time-course analysis of samples of tetraubiquitin chains incubated with PINK1 kinase and ATP and processed with SDS-PAGE.
15. Rabbit polyclonal anti-VDAC-1 (Abcam, Ab15895): Validated by the manufacturer for western blotting in extracts from subcellular fractionation and from several species (PMID: 37758019; PMID: 38071198)
16. Rabbit monoclonal anti-vinculin (Abcam, Ab219649): Validated by the manufacturer for western blotting in Vinculin cell line knockout.
17. Total OXPHOS Blue Native WB Antibody Cocktail (1:1000, Abcam, Ab110412): Validated by the manufacturer in two dimensions Blue Native PAGE analysis of fibroblasts that are normal or complex I deficient. Cited in several studies (PMID: 38504132; PMID: 4457891)

Eukaryotic cell lines

Policy information about cell lines and Sex and Gender in Research

Cell line source(s)	Jurkat cells (ATCC, TIB-152), Mouse Adult Fibroblasts from Mito-QC mice (Dundee University).
Authentication	Mouse Adult Fibroblasts were verified by testing for fibroblast markers.
Mycoplasma contamination	All cell lines were regularly tested for mycoplasma contamination and tested negative throughout the study.
Commonly misidentified lines (See ICLAC register)	No commonly misidentified lines were used in this study

Animals and other research organisms

Policy information about studies involving animals; ARRIVE guidelines recommended for reporting animal research, and Sex and Gender in Research

Laboratory animals	A novel zebrafish transgenic line was registered at the central repository ZFIN.org under the designation Tg(actc1b:ZsGreen-map1lc3;cryaa:TdTomato)nei014 and can be found on www.zfin.org in the section “Tg/Mutants” entering the identifier nei014 in the search field. Wild-type zebrafish were used from a common AB line derived from multiple laboratories andand origin from stock centers.<br>10 weeks old C57BL/6J mice (Janvier, C57BL/6JRj) and 8-months-old SAMP8 AKR/J 8(Envigo) males were studied.<br>For C.elegans studies the strain used in this study was the wild type Bristol N2,, CF1038 daf-16(mu86), RB2547 pink-1(ok3538), and QV225 skn-1(zj15). All strains were obtained from the Caenorhabditis Genetics Center.
Wild animals	No wild animals were used in this study
Reporting on sex	Only male mice could be used for this study due to availability constrains from commercial provider for aged animals necessary for this study.
Field-collected samples	No field samples were collected
Ethics oversight	All experiments adhered to Swiss regulation on animal experimentation and European Community Council Directive 2010/63/EU. All procedures were approved by the Nestlé Ethical Committee (ASP-16-38-EXT), the Office Vétérinaire Cantonal Vaudois (VD2770, VD3195 and VD3484) and the General Direction of Animal Health and Veterinary Drugs of the Italian Ministry of Health with authorizations n. 924/2021-PR and 885/2020-PR.

Note that full information on the approval of the study protocol must also be provided in the manuscript.

Plants

Seed stocks	Not applicable
Novel plant genotypes	Not applicable
Authentication	Not applicable

Flow Cytometry

Plots

Confirm that:

☒ The axis labels state the marker and fluorochrome used (e.g. CD4-FITC).

☒ The axis scales are clearly visible. Include numbers along axes only for bottom left plot of group (a 'group' is an analysis of identical markers).

☒ All plots are contour plots with outliers or pseudocolor plots.

☒ A numerical value for number of cells or percentage (with statistics) is provided.

Methodology

Sample preparation	Autophagy in Jurkat cells has been measured by flow cytometry using Guava LC3 antibody-based assay kit (Luminex) following the manufacturer's instruction. The kit contains reagent A (inhibitor of lysosomal activity), reagent B (permeabilization solution) and an anti-LC3 antibody conjugated with the fluorophore Fluoresceine Isothiocyanate (FITC) (clone 4E-12). Cells in a 96 V bottom plate have been permeabilized with reagent B to remove the cytoplasmic form of LC3 (LC3-I) incubated with the FITC conjugated LC3 antibody prior acquisition
Instrument	Becton Dickinson LSORP Fortessa analyzer
Software	FCS Express Software (De Novo Software)
Cell population abundance	100.000 cells / condition
Gating strategy	All Flow cytometry based expriments were performed with fluorescent markers following manufacturer's instructions quantifying mean intensities and, as indicated in the methods section, ratios of absorption spectra within a single cell population. Gating was performed on size and granularity, single cells and live cells using DAPI. Gating for autophagy flux, JC10 and MitoSox were done based on the following procedure: A first gate was drawn including single cell populations and to exclude out-of-scale events (Singlet). Then a Scatter Gate was drawn to select cell population in a forward/side scatter (FSC/SSC) plot. Cell population in a forward/side scatter (FSC/SSC) plot. MFI values for the LC3 marker were obtained by histogram analysis in the gate "Scatter" (right panel, histograms for control (DMSO) and treated cells with thymol 250µM. Gating for JC10 and MitoSox followed the same gating and were quantified through mean fluorescence intensity according to the respective spectra.

☒ Tick this box to confirm that a figure exemplifying the gating strategy is provided in the Supplementary Information.
