# Supplementary figures and images for: Herbal terpenoids activate autophagy and mitophagy through modulation of bioenergetics and protect from metabolic stress, sarcopenia and epigenetic aging
Source: Nat Aging. 2025 Sep 24;5(10):2003–21. doi: 10.1038/s43587-025-00957-4 (PMC12532568; doi:10.1038/s43587-025-00957-4)

Source data Extended data figure 1d

p-S6 blot

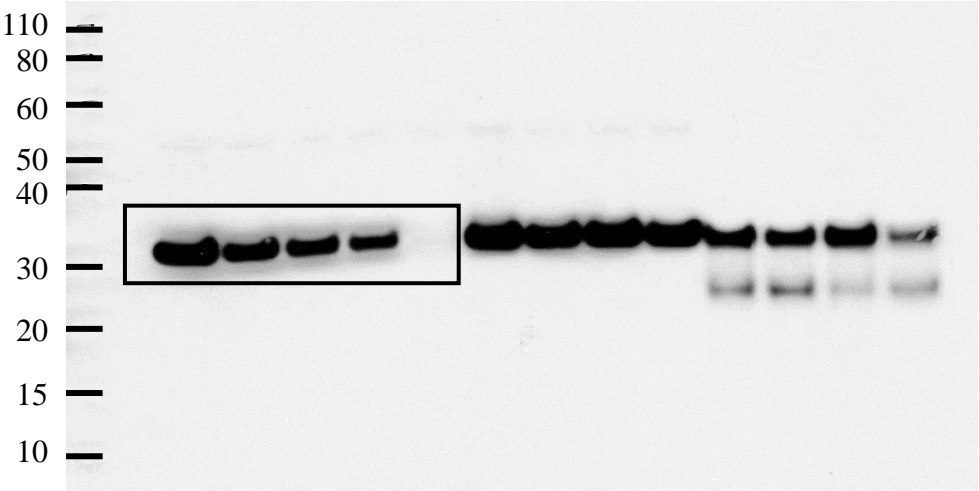

GAPDH blot

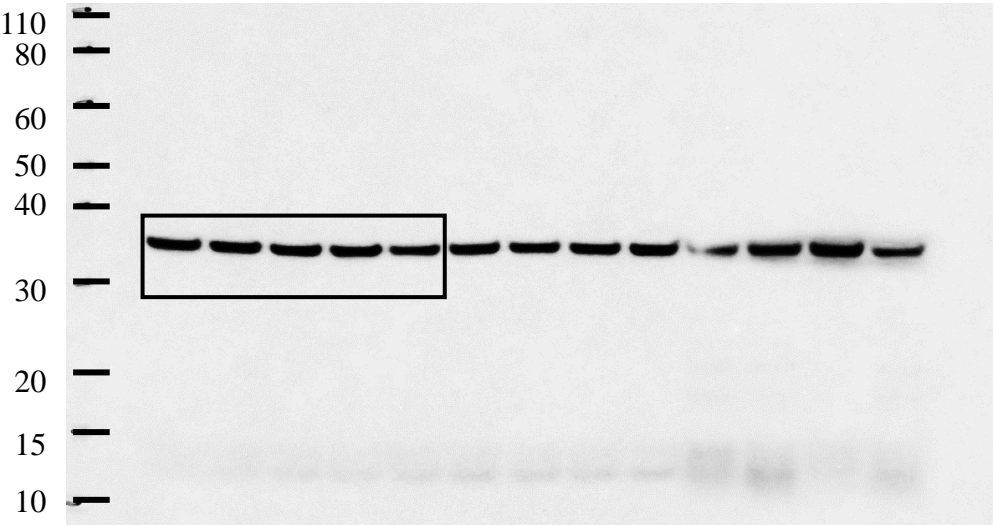

S6 blot

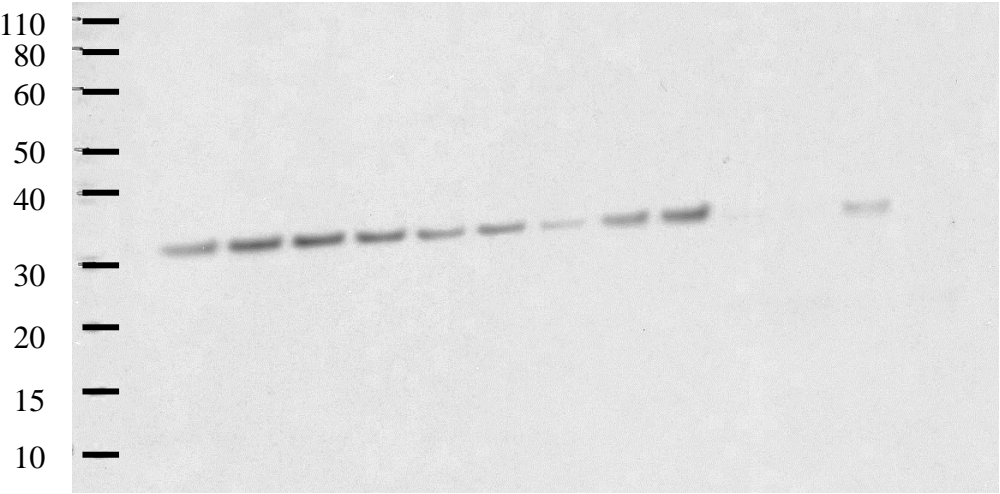

Supplement: Supplementary file 4 — Uncropped western blots. [file 43587_2025_957_MOESM4_ESM.pdf]

## Source data figure 7k

PINK1 blot

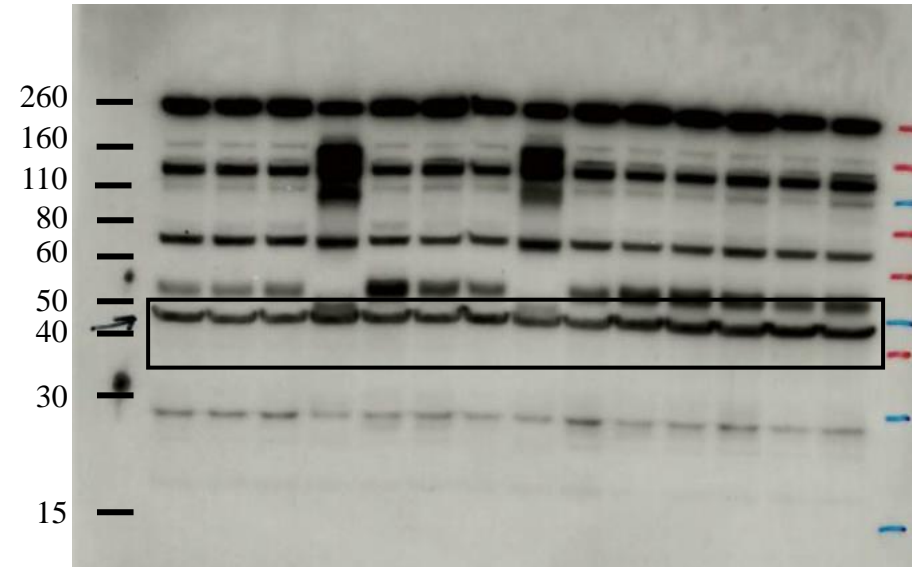

VINCULIN blot

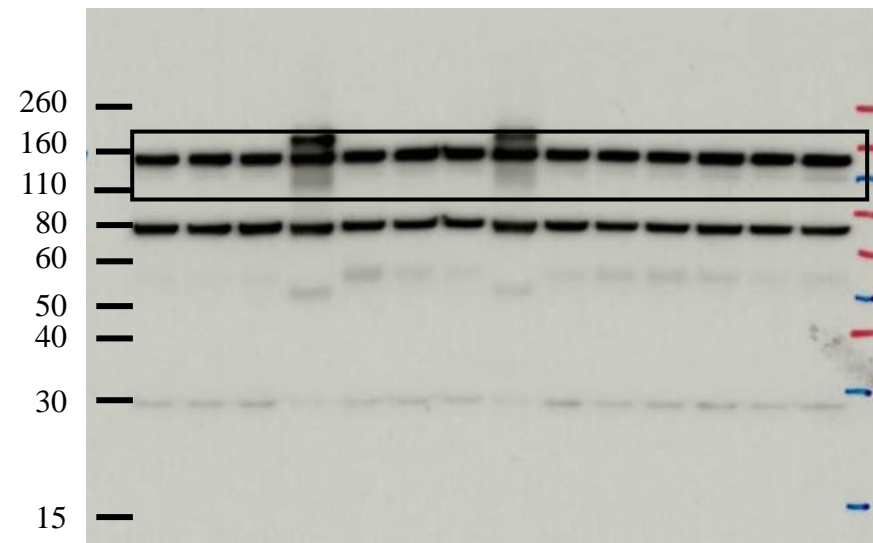

Supplement: Supplementary file 8 — Uncropped western blots. [file 43587_2025_957_MOESM8_ESM.pdf]
