# Supplementary material for: Herbal terpenoids activate autophagy and mitophagy through modulation of bioenergetics and protect from metabolic stress, sarcopenia and epigenetic aging
Source: Nat Aging. 2025 Sep 24;5(10):2003–21. doi: 10.1038/s43587-025-00957-4 (PMC12532568; doi:10.1038/s43587-025-00957-4)

Source data Extended data figure 2d-e

p-S6 blot

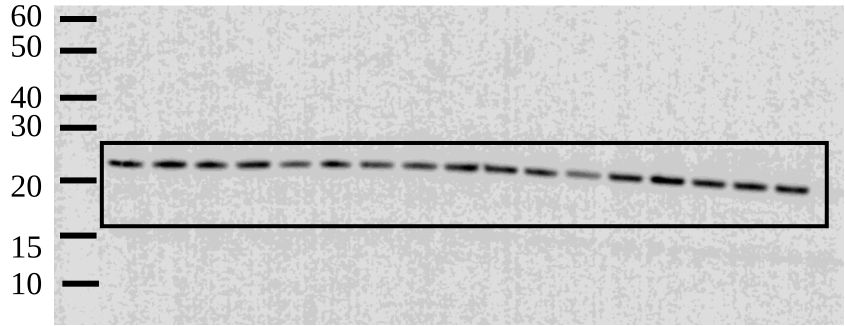

S6 blot

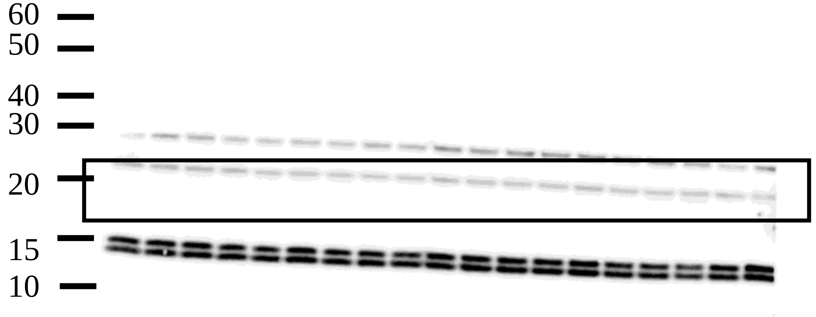

β-TUBULIN blot

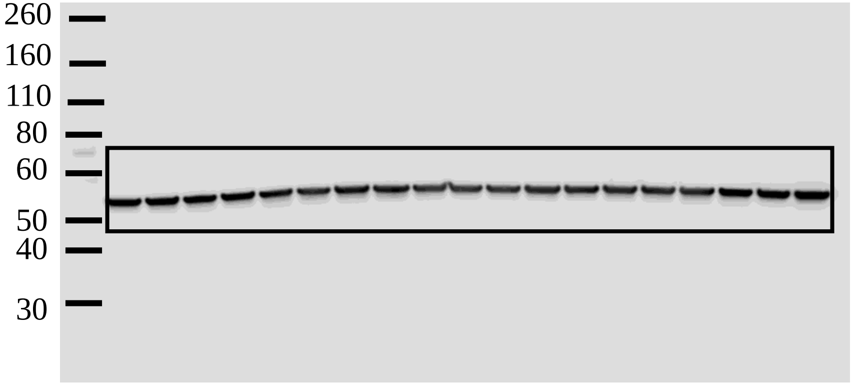

p-AMPK blot

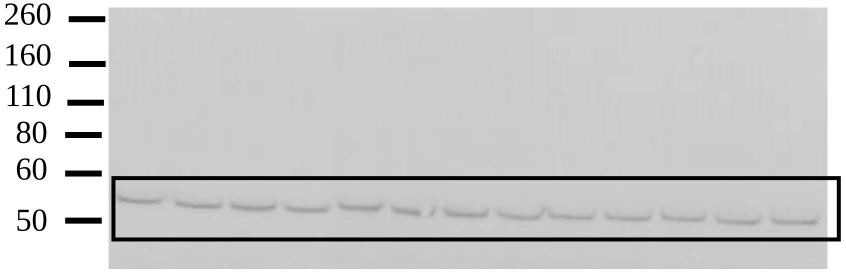

AMPK blot

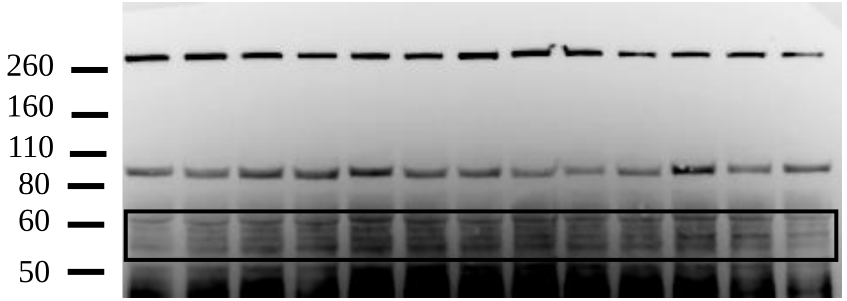

β-TUBULIN blot

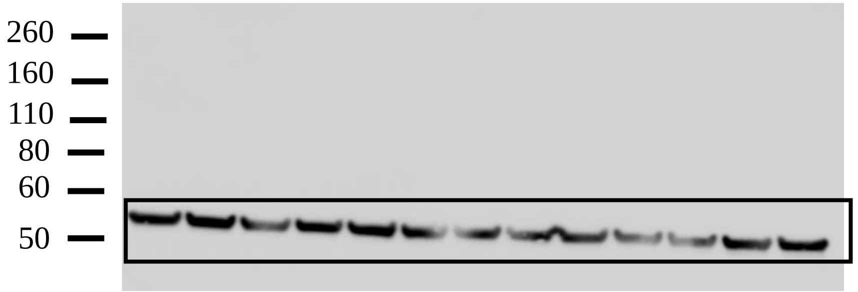

Supplement: Supplementary file 5 — Uncropped western blots. [file 43587_2025_957_MOESM5_ESM.pdf]
