# Supplementary material for: Herbal terpenoids activate autophagy and mitophagy through modulation of bioenergetics and protect from metabolic stress, sarcopenia and epigenetic aging
Source: Nat Aging. 2025 Sep 24;5(10):2003–21. doi: 10.1038/s43587-025-00957-4 (PMC12532568; doi:10.1038/s43587-025-00957-4)

Source data Figure 5

LC3 blot (fig. 5b)

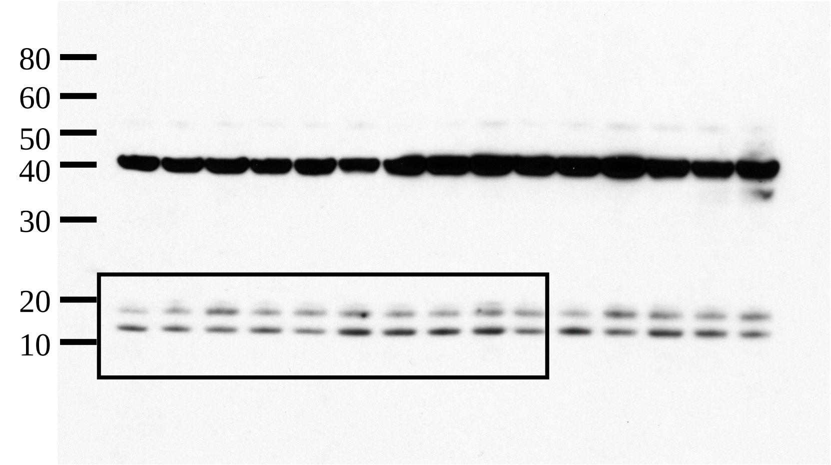

HSC70 blot (fig 5b)

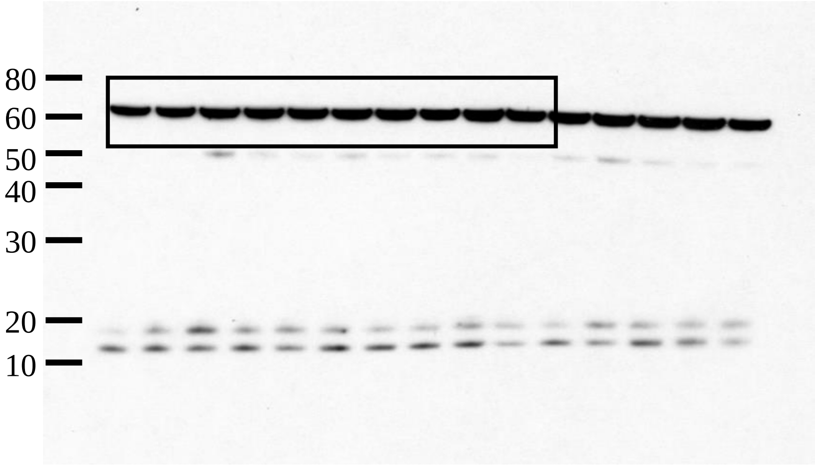

P62 blot (fig 5b)

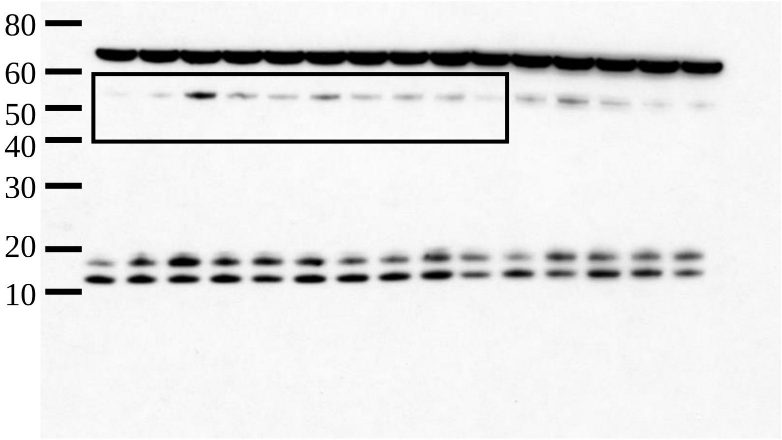

LC3 blot (fig 5i)

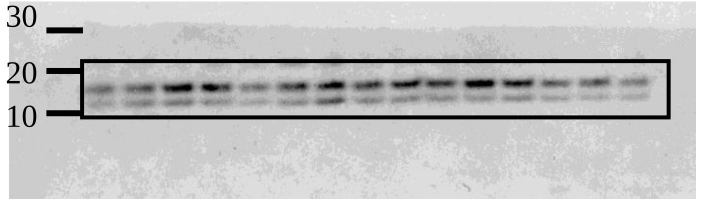

P-S65 Ubiquitin (fig 5b)

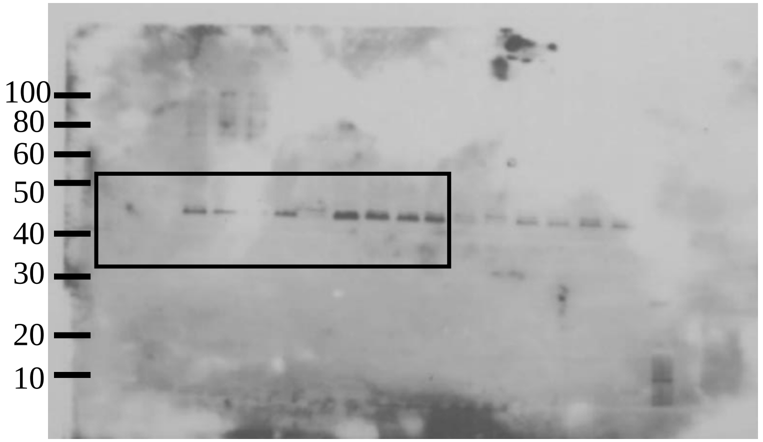

VDAC (fig 5b)

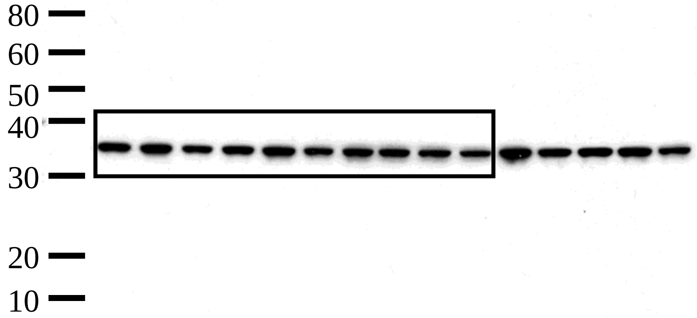

P62-HSC70 blot (fig 5i)

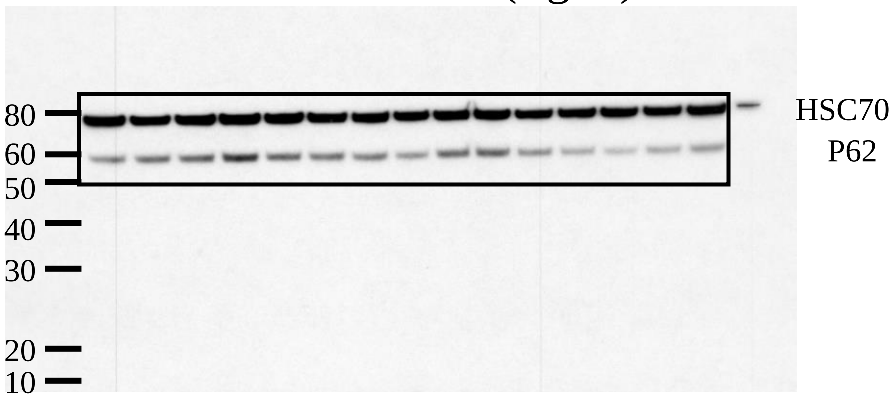

Supplement: Supplementary file 7 — Uncropped western blots. [file 43587_2025_957_MOESM7_ESM.pdf]
